# Deep learning-based 3D quantitative total tumor burden predicts early recurrence of BCLC A and B HCC after resection

**DOI:** 10.1007/s00330-024-10941-y

**Published:** 2024-07-19

**Authors:** Hong Wei, Tianying Zheng, Xiaolan Zhang, Chao Zheng, Difei Jiang, Yuanan Wu, Jeong Min Lee, Mustafa R. Bashir, Emily Lerner, Rongbo Liu, Botong Wu, Hua Guo, Yidi Chen, Ting Yang, Xiaoling Gong, Hanyu Jiang, Bin Song

**Affiliations:** 1 Department of Radiology, Functional, and Molecular Imaging Key Laboratory of Sichuan Province, West China Hospital, Sichuan University, Chengdu, Sichuan 610041 China; 2 Department of Radiology, Seoul National University Hospital, Seoul, 03080 Republic of Korea; 3 Shukun Technology Co., Ltd, Beijing, 100102 China; 4 Big Data Research Center, University of Electronic Science and Technology of China, Chengdu, Sichuan 610000 China; 5 Department of Radiology, Seoul National University College of Medicine, Seoul, 03080 Republic of Korea; 6 Department of Radiology, Duke University Medical Center, Durham, NC 27710 USA; 7 Center for Advanced Magnetic Resonance in Medicine, Duke University Medical Center, Durham, NC 27705 USA; 8 Division of Gastroenterology, Department of Medicine, Duke University Medical Center, Durham, NC 27710 USA; 9 Center for Biomedical Imaging Research, Department of Biomedical Engineering, School of Medicine, Tsinghua University, Beijing, 100102 China; 10 Department of Radiology, Sanya People’s Hospital, Sanya, Hainan 572000 China

**Keywords:** Carcinoma (hepatocellular), Tumor burden, Recurrence, Magnetic resonance imaging, Hepatectomy

## Abstract

**Objectives:**

This study aimed to evaluate the potential of deep learning (DL)-assisted automated three-dimensional quantitative tumor burden at MRI to predict postoperative early recurrence (ER) of hepatocellular carcinoma (HCC).

**Materials and methods:**

This was a single-center retrospective study enrolling patients who underwent resection for BCLC A and B HCC and preoperative contrast-enhanced MRI. Quantitative total tumor volume (cm^3^) and total tumor burden (TTB, %) were obtained using a DL automated segmentation tool. Radiologists’ visual assessment was used to ensure the quality control of automated segmentation. The prognostic value of clinicopathological variables and tumor burden-related parameters for ER was determined by Cox regression analyses.

**Results:**

A total of 592 patients were included, with 525 and 67 patients assigned to BCLC A and B, respectively (2-year ER rate: 30.0% vs. 45.3%; hazard ratio (HR) = 1.8; *p* = 0.007). TTB was the most important predictor of ER (HR = 2.2; *p* < 0.001). Using 6.84% as the threshold of TTB, two ER risk strata were obtained in overall (*p* < 0.001), BCLC A (*p* < 0.001), and BCLC B (*p* = 0.027) patients, respectively. The BCLC B low-TTB patients had a similar risk for ER to BCLC A patients and thus were reassigned to a BCLC A_n_ stage; whilst the BCLC B high-TTB patients remained in a BCLC B_n_ stage. The 2-year ER rate was 30.5% for BCLC A_n_ patients vs. 58.1% for BCLC B_n_ patients (HR = 2.8; *p* < 0.001).

**Conclusions:**

TTB determined by DL-based automated segmentation at MRI was a predictive biomarker for postoperative ER and facilitated refined subcategorization of patients within BCLC stages A and B.

**Clinical relevance statement:**

Total tumor burden derived by deep learning-based automated segmentation at MRI may serve as an imaging biomarker for predicting early recurrence, thereby improving subclassification of Barcelona Clinic Liver Cancer A and B hepatocellular carcinoma patients after hepatectomy.

**Key Points:**

- *Total tumor burden (TTB) is important for Barcelona Clinic Liver Cancer (BCLC) staging, but is heterogenous.*
- *TTB derived by deep learning-based automated segmentation was predictive of postoperative early recurrence.*
- *Incorporating TTB into the BCLC algorithm resulted in successful subcategorization of BCLC A and B patients.*

## Introduction

Hepatocellular carcinoma (HCC) is the sixth most common malignancy and the third leading cause of cancer-related deaths worldwide [1]. Surgical resection is the mainstay curative treatment for patients with resectable HCC [2], and local ablation is the potentially curative therapy for small early-stage HCC [3]. While patients with early-stage HCC are considered the optimal surgical candidates, as per the Barcelona Clinic Liver Cancer (BCLC) algorithm [4], resection for well-selected intermediate-stage (BCLC B) HCC has been associated with improved survival compared to transarterial chemoembolization (TACE) [5, 6]. Therefore, identifying individuals with BCLC B HCC who would benefit from hepatectomy is pivotal for personalized clinical decision-making.

Tumor burden is a critical factor in determining staging and treatment plans, yet it exhibits considerable heterogeneity within the BCLC stages A and B. As a result, patients assigned to the same BCLC stage may have distinct prognoses while those at different stages might demonstrate similar survival outcomes [2, 7, 8]. One potential explanation is that a one-dimensional measurement is used in the BCLC algorithm to estimate tumor burden, which may under- or overestimate tumor size due to its inaccuracies. Despite being easy-to-use and highly reproducible, this one-dimensional approach might be insufficient to profile the complex morphology of three-dimensional (3D) tumors. Thus, identifying more accurate approaches for tumor burden evaluation is key to optimizing patient risk stratification and refining treatment allocations.

The 3D volumetric analysis may offer a promising solution for accurate tumor burden quantification. Previous research has indicated that the MRI-based 3D quantitative tumor burden demonstrates superior capability compared to one-dimensional measurements for assessing prognosis and treatment response in HCC patients [9–12]. However, these studies were limited by small sample sizes (ranging from 78 to 162 patients) and the utilization of semi-automated segmentation. Although these semi-automated methods have mitigated some challenges associated with manual segmentation, including time consumption, labor intensity, and dependence on operators’ experience, they still involve user input (i.e., manual adjustment of inaccurate segmentations) and may suffer from inter-reader variability. Fortunately, the recently implemented artificial intelligence (AI) deep-learning (DL) algorithms for segmentation have allowed automated quantification of liver and HCC volumes [13–16]. This approach has the potential to improve both efficiency and reproducibility over traditional methodologies. Nonetheless, to our knowledge, data remain scarce on the potential of automated segmentation-based volumetric analyses in the prognostication of BCLC A and B HCC following resection.

This study aimed to evaluate the utility of MRI-based 3D quantitative tumor burden using automated DL segmentation algorithms in predicting early recurrence (ER) among patients undergoing curative resection for BCLC A and B HCC. We also assessed whether 3D quantitative tumor burden could be used to subcategorize these patients for more effective prognostication. Radiologists’ visual assessment was used to ensure the quality control of automated segmentation.

## Materials and methods

This single-institution, retrospective study was approved by our institutional review board, and the requirement for informed consent was waived.

### Patients

**Fig. 1:**
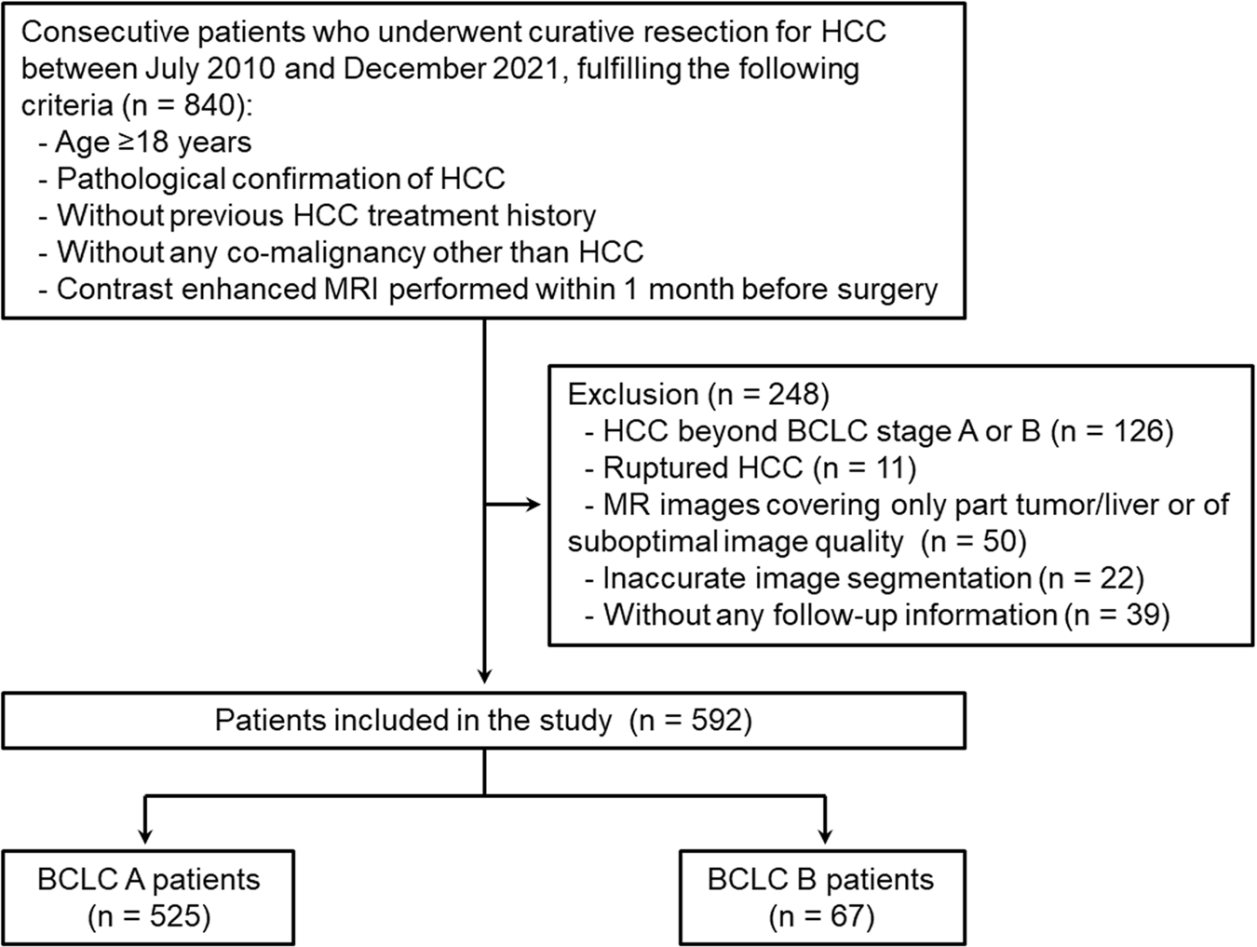
Study flowchart. BCLC, Barcelona Clinic Liver Cancer; HCC, hepatocellular carcinoma; MRI, magnetic resonance imaging

Consecutive patients who underwent curative resection for HCC between July 2010 and December 2021 were retrospectively included in this study if they fulfilled the following criteria: *(a)* age ≥ 18 years, *(b)* pathological confirmation of HCC, *(c)* without previous HCC treatment history, *(d)* without any co-malignancy other than HCC, and *(e)* contrast-enhanced MRI performed within 1 month before surgery. The exclusion criteria were *(a)* HCC beyond BCLC stage A or B, *(b)* ruptured HCC, *(c)* MR images covering only part of the tumor/liver or of suboptimal image quality (e.g., severe artifacts), *(d)* inaccurate image segmentation (detailed below), and *(e)* without any follow-up information (Fig. 1).

Baseline patient characteristics were collected from electronic medical records. The diagnosis of cirrhosis was established based on the Japanese Society of Gastroenterology/Japanese Society of Hepatology joint Clinical Practice Guidelines [17]. Clinical decisions for surgical resection were based on discussions of liver surgery expert panels while fully considering patient performance status, co-morbidities, liver function reserve, estimated future liver remnant volume, and tumor extent. Briefly, surgical resection was considered for patients with localized HCC and preserved liver function in the absence of clinically significant portal hypertension [1, 18, 19]. For patients with borderline tumors (e.g., R0 resection was technically challenging, or remnant liver volume was anticipated to be around 30% in non-cirrhotic patients or 40% in cirrhotic patients), the selection among surgical resection and other alternatives such as locoregional therapies was determined based on multidisciplinary discussions. Notably, some patients received postoperative adjuvant therapies (e.g., TACE, systemic therapy, and radiotherapy), as in our practice adjuvant therapies have been routinely recommended since 2017 for patients at anticipated high-risk of recurrence (e.g., multiple tumors, tumor size > 5 cm, Edmondson grade 3–4, and microvascular invasion (MVI)) [20, 21].

Of note, 222 patients have been reported previously [22]. While the prior work proposed a preoperative prognostic score for overall survival, the current work focused on exploring 3D quantitative tumor burden biomarkers for predicting postsurgical ER of HCC.

### MRI acquisition

MRI was performed with various 3.0-T or 1.5-T systems. The choice between extracellular and hepatobiliary MRI contrast agents was at the surgeons’ discretion or based on the multidisciplinary team’s recommendations according to our institutional standard [23]. MRI systems and acquisition protocols are described in Supplementary Material 1 and Table S1.

### Image analysis

#### One-dimensional measurement

One-dimensional measurement of tumors was performed by two abdominal radiologists (H.W. and H.Y.J., with 5 and 8 years of experience in liver MRI, respectively) who were aware that all patients had HCCs but were blinded to other information. Total tumor size (TTS) was defined as the sum of the size of all HCC lesions. Additionally, the single tumor > 7 cm and multiple tumors beyond up-to-seven criteria were assessed to reassign the BCLC staging [8]. Details of one-dimensional measurement are presented in Supplementary Material 2.

#### Three-dimensional analysis

**Fig. 2:**
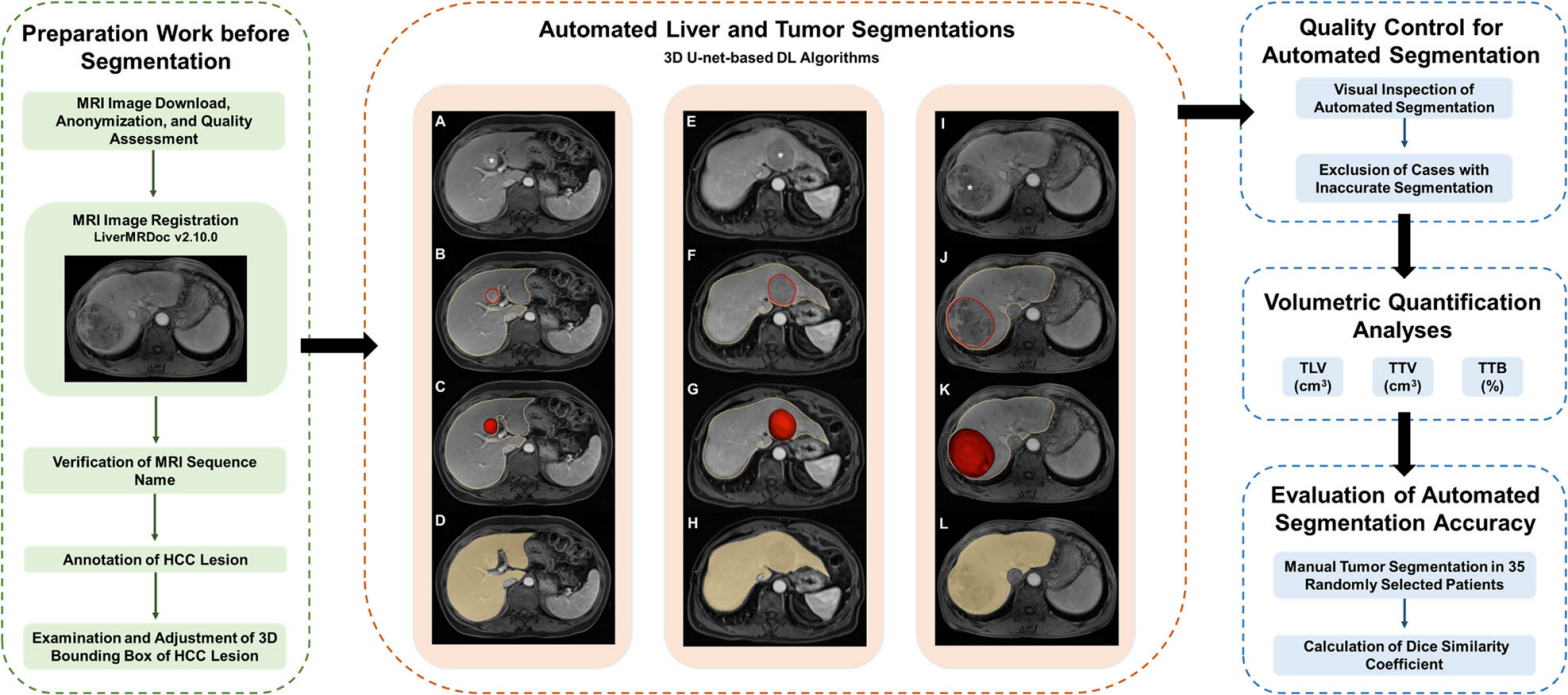
Workflow of automated segmentation and volumetric quantification analyses. **A**–**L** Three representative cases of automated segmentation. **A**, **E**, **I** Axial T1-weighted portal-venous phase images in 3 patients show (**A**) a 2.5 cm HCC (*) in segment IV, (**E**) a 5.8 cm HCC (*) in segments II and III, and (**I**) an 8.8 cm HCC (*) in segments V–VIII. **B**, **F**, **J** Automated tumor (red lines) and liver (yellow lines) segmentations to create corresponding segmentation masks. **C**, **G**, **K** The 3D segmentation masks in red represent the total tumor volumes, which were (**C**) 9.15 cm^3^, (**G**) 74.57 cm^3^, and (**K**) 292.88 cm^3^, respectively. **D**, **H**, **L** The 3D segmentation masks in yellow represent total liver volumes, which were (**D**) 1214.55 cm^3^, (**H**) 1010.10 cm^3^, and (**L**) 1477.59 cm^3^, respectively. DL, deep learning; HCC, hepatocellular carcinoma; MRI, magnetic resonance imaging; TLV, total liver volume; TTB, total tumor burden; TTV, total tumor volume; 3D, three-dimensional

The 3D automated image segmentation and volumetric quantification analyses were performed using commercial visualization and analysis software (LiverMRDoc; version 2.10.0; SHUKUN) (Fig. 2). Deep-learning algorithms for automated segmentation and volumetric quantification are detailed in Supplementary Material 3 and Fig. S1.

#### Preparation work before segmentation

Before initiating automated segmentation, one radiologist (H.W.) inspected the MR images and verified the sequence names, the HCC lesions, and the corresponding 3D bounding boxes (i.e., the automated lesion detection annotation) on the AI software platform. For patients (*n* = 25) with inaccurate 3D bounding boxes (e.g., failing to detect HCC lesions or delineating the whole tumors), manual adjustment was conducted to achieve accurate localization of tumors.

#### Automated liver and tumor segmentation

Automated segmentation of liver and HCC lesions was performed on each sequence by 3D U-net-based DL algorithms [24]. Briefly, a volumetric segmentation mask derived from portal-venous phase images was used to quantify whole liver volume (with intrahepatic vasculature and focal liver lesions included). A volumetric segmentation mask obtained on the optimal sequence automatedly determined by the AI software was used to quantify tumor volume. For a total of 716 tumors included in this study, tumor volumes were extracted from portal-venous phase (*n* = 617), delayed phase (*n* = 33), hepatobiliary phase (*n* = 32), precontrast phase (*n* = 17), arterial phase (*n* = 8), opposed phase (*n* = 3), in-phase (*n* = 2), T2-weighted imaging (*n* = 2), transitional phase (*n* = 1), diffusion-weighted imaging (*n* = 1), respectively.

#### Quality control for automated segmentation

Two radiologists (H.W. and T.Y.Z.) independently and visually inspected the segmented tumors and liver for each patient, with discrepancies resolved by discussion to reach a consensus. Cases with inaccurate segmentations were excluded from the volumetric analyses. Manual adjustment was not performed because this study aimed to focus on the prognostic potential of this automated technique.

#### Volumetric quantification analyses

Volumetric quantification analyses were performed to calculate total liver volume (TLV, cm^3^), total tumor volume (TTV, cm^3^), and total tumor burden (TTB, %). Specifically, TTV was defined as the sum of the volume of all HCC lesions. TTB was defined as the ratio of TTV to the TLV as follows: TTB = TTV/TLV × 100%.

#### Evaluation of automated segmentation accuracy

To evaluate the accuracy of automated DL segmentation, we randomly selected 35 patients (with 30 single HCCs and 5 multiple HCCs) from the final included cohort for manual tumor segmentation. One radiologist (H.W.), who was blinded to the automated segmentation results, manually segmented tumors using ITK-SNAP (version 3.8.0; www.itksnap.org).

### Patient follow-up

Follow-up protocols included serum alpha-fetoprotein (AFP) level, liver function tests, and contrast-enhanced ultrasound, computed tomography, or MRI performed 1 month after surgical resection, every 3 months during the first 2 years, and every 6 months subsequently. Patients were followed up until death or the end date of this study (May 1, 2022). ER was defined as tumor recurrence within the first 2 years after surgery.

### Statistical analysis

Categorical variables were compared using the Chi-squared test or Fisher’s exact test, while continuous variables were compared using the Student’s *t*-test or Mann–Whitney *U*-test, as appropriate. Inter-reader agreement for one-dimensional measurement was assessed by intraclass correlation coefficient (ICC). Consistency between automated and manual tumor segmentations was evaluated by the dice similarity coefficient (DSC).

The prognostic value of clinical-radiological-pathological characteristics to predict ER was assessed by uni- and multivariable Cox regression analyses. Variables with *p* < 0.1 at univariable analyses were included in the multivariable analysis using a backward stepwise approach based on the akaike information criterion and five-fold cross-validation.

Survival curves were plotted by the Kaplan–Meier method and compared by the log-rank test. X-tile plots provide a single and intuitive approach for evaluating the association between variables and survival. Given that no well-recognized thresholds have been established for TTS, TTV, and TTB, X-tile plots were used to determine the optimal cutoffs of these variables based on the highest *χ*² value (minimum *p*-value) defined by Kaplan–Meier survival analysis and log-rank test. X-tile plots were generated by X-tile software (version 3.6.1; Yale University School of Medicine) [25]. A bootstrap resampling (*n* = 1000) technique was used to test the robustness of survival risk stratification based on BCLC and modified BCLC systems.

Statistical analyses were performed with R (version 4.3.0; The R Foundation for Statistical Computing), Python (version 3.9.1; https://www.python.org/), and IBM SPSS software (version 26.0; SPSS Inc.). A two-tailed *p* < 0.05 was considered statistically significant.

## Results

### Patient characteristics

A total of 592 patients (median age, 54 years; interquartile range (IQR), 46–62 years; 517 men) were included, with 525 (88.7%) and 67 (11.3%) classified in BCLC stage A and B, respectively. There were 333 patients (56.3%) classified as China Liver Cancer stage Ia, 192 (32.4%) as Ib, 59 (10.0%) as IIa, and 8 (1.4%) as IIb. Overall, 17.6% (104/592) of patients underwent postoperative adjuvant therapies. ER occurred in 28.9% (171/592) of patients during a median follow-up period of 52.4 months (IQR, 29.5-78.5 months). Patient characteristics are summarized in Table 1.

**Table 1:** Patient characteristics

Characteristic	Total (*n* = 592)
Age, years^a^	54 (46–62)
Sex	
Female	75 (12.7)
Male	517 (87.3)
Underlying liver disease	
HBV	573 (96.8)
Other	19 (3.2)
Cirrhosis	308 (52.0)
Child-Pugh class	
A	585 (98.8)
B	7 (1.2)
AFP, ng/mL	
< 400	452 (76.4)
≥ 400	140 (23.6)
BCLC stage	
A	525 (88.7)
B	67 (11.3)
CNLC stage	
Ia	333 (56.3)
Ib	192 (32.4)
IIa	59 (10.0)
IIb	8 (1.4)
Milan criteria	
Within	353 (59.6)
Beyond	239 (40.4)
Postoperative adjuvant therapy	104 (17.6)
TACE	58 (9.8)
Systemic therapy	34 (5.7)
TACE + systemic therapy	9 (1.5)
Radiotherapy	3 (0.5)
Contrast agent type of MRI	
ECA	400 (67.6)
HCA	192 (32.4)
Total liver volume, cm^3 a^	1246.82 (1086.57–1421.06)
Tumor characteristics	
Tumor multiplicity	
Unifocal	505 (85.3)
Multifocal	87 (14.7)
TTS, cm^a^	4.3 (3.2–6.6)
TTV, cm^3 a^	22.09 (8.84–72.20)
TTB, %^a^	1.82 (0.72–6.20)
Single tumor > 7 cm and multiple tumors beyond up-to-seven criteria	
Reassigned BCLC A	459 (77.5)
Single tumor > 2 and ≤ 7 cm	416 (70.3)
Multiple tumors within up-to-seven criteria	43 (7.3)
Reassigned BCLC B	133 (22.5)
Single tumor > 7 cm	89 (15.0)
Multiple tumors exceeding up-to-seven criteria	44 (7.4)
Tumor differentiation^b^	
Well or moderate	398 (67.8)
Poor	189 (32.2)
MVI^b^	
Absent	204 (52.4)
Present	185 (47.6)

Unless indicated otherwise, data are the number of patients, with percentages in parentheses

*AFP* alpha-fetoprotein, *BCLC* Barcelona Clinic Liver Cancer, *CNLC* China Liver Cancer, *ECA* extracellular contrast agent, *HBV* hepatitis B virus, *HCA* hepatobiliary contrast agent, *MRI* magnetic resonance imaging, *MVI* microvascular invasion, *TACE* transarterial chemoembolization, *TTB* total tumor burden, *TTS* total tumor size, *TTV* total tumor volume

^a^ Data are medians, with interquartile range in parentheses

^b^ There were 5 and 203 missing values for tumor differentiation and MVI in the entire study cohort, respectively

### Inaccurate image segmentations

**Fig. 3:**
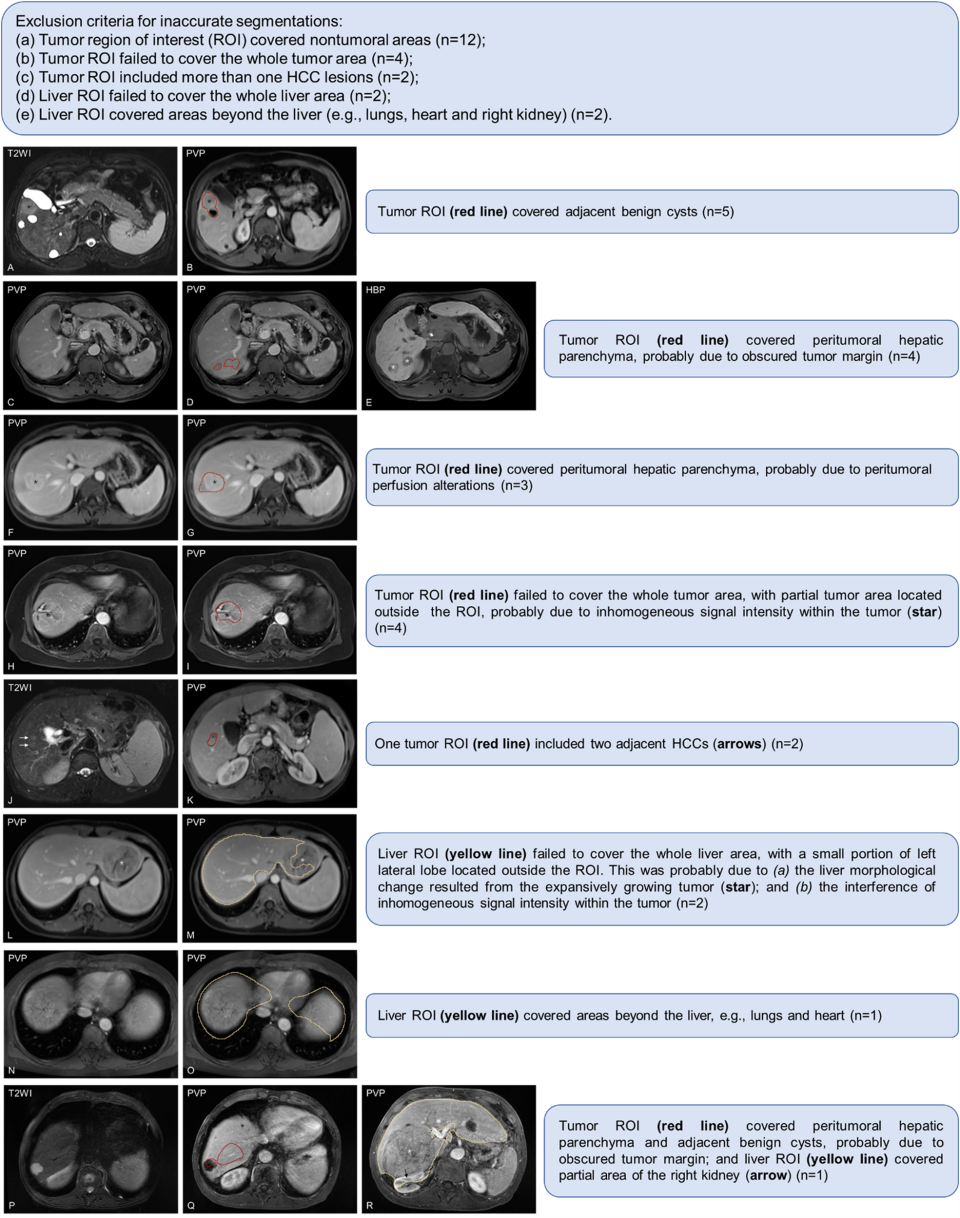
**A**–**R** Examples of inaccurate image segmentations. HBP, hepatobiliary phase; HCC, hepatocellular carcinoma; PVP, portal-venous phase; ROI, region of interest; T2WI, T2-weighted imaging

Twenty-two cases with inaccurate liver and/or tumor segmentations were excluded from the volumetric analyses. Examples of inaccurate segmentations are shown in Fig. 3.

### Evaluation of inter-reader agreement and segmentation accuracy

The inter-reader agreement was excellent for TTS (ICC = 0.987; 95% confidence interval (CI): 0.983, 0.990). For the 40 HCCs (median size, 4.2 cm; IQR, 3.0–7.3 cm) in 35 randomly selected patients, the mean DSC between automated and manual tumor segmentations was 0.85 ± 0.11 (median, 0.88; IQR, 0.82–0.92) on all sequences. DSCs for each sequence are detailed in Table S2 and Fig. S2.

### Predictors for ER based on Cox regression analyses

#### Entire cohort

For the entire cohort (*n* = 592), 8 parameters, including serum AFP level, postoperative adjuvant therapy, BCLC stage, tumor multiplicity, TTS, TTV, TTB, and the single tumor > 7 cm, and multiple tumors beyond up-to-seven criteria, were significantly associated with ER at univariable Cox regression analyses (*p* < 0.05 for all) (Table 2). On subsequent multivariate Cox regression analysis, serum AFP level (hazard ratio (HR) = 1.4; 95% CI: 1.0, 1.9; *p* = 0.05), tumor multiplicity (HR = 1.8; 95% CI: 1.2, 2.6; *p* = 0.003) and TTB (HR = 2.2; 95% CI: 1.6, 3.0; *p* < 0.001) were found to be predictive of ER (Table 2). The C-index for TTB in predicting the risk of ER was 0.589 (95% CI: 0.553, 0.625).

**Table 2:** Predictors for early recurrence based on Cox regression analyses

Variable	All patients (*n* = 592)				Patients with available pathological data (*n* = 385)			
	Univariable analysis		Multivariable analysis		Univariable analysis		Multivariable analysis	
	Hazard ratio	*p-*value	Hazard ratio	*p-*value	Hazard ratio	*p-*value	Hazard ratio	*p*-value
Age, years (< 65 vs. ≥ 65)	0.7 (0.5, 1.1)	0.14	…	…	0.6 (0.4, 1.0)	0.07		
Sex (female vs. male)	1.1 (0.7, 1.7)	0.81	…	…	0.9 (0.5, 1.6)	0.76	…	…
Underlying liver disease (HBV vs. non-HBV)	1.0 (0.4, 2.5)	0.93	…	…	0.9 (0.3, 2.5)	0.88	…	…
Cirrhosis (absent vs. present)	1.0 (0.8, 1.4)	0.78	…	…	0.9 (0.6, 1.3)	0.59	…	…
Child-Pugh class (A vs. B)	2.0 (0.6, 6.3)	0.24	…	…	1.8 (0.4, 7.2)	0.42	…	…
AFP, ng/mL (< 400 vs. ≥ 400)	1.5 (1.1, 2.0)	0.02	1.4 (1.0, 1.9)	0.05	2.0 (1.4, 2.9)	< 0.001	1.7 (1.2, 2.5)	0.007
Postoperative adjuvant therapy (absent vs. present)	1.5 (1.1, 2.2)	0.02	…	…	1.4 (1.0, 2.2)	0.07	…	…
BCLC stage (A vs. B)	1.8 (1.2, 2.7)	0.008	…	…	1.7 (1.0, 2.9)	0.04	…	…
Tumor multiplicity (unifocal vs. multifocal)	1.8 (1.2, 2.6)	0.002	1.8 (1.2, 2.6)	0.003	1.6 (1.0, 2.6)	0.04	1.6 (1.0, 2.5)	0.06
TTS, cm (< 4.1 vs. ≥ 4.1)	2.2 (1.6, 3.1)	< 0.001	…	…	2.4 (1.6, 3.7)	< 0.001	…	…
TTV, cm^3^ (< 85.09 vs. ≥ 85.09)	2.1 (1.6, 2.9)	< 0.001	…	…	2.4 (1.7, 3.5)	< 0.001	…	…
TTB, % (< 6.84 vs. ≥ 6.84)	2.3 (1.6, 3.1)	< 0.001	2.2 (1.6, 3.0)	< 0.001	2.5 (1.8, 3.7)	< 0.001	2.0 (1.4, 3.0)	< 0.001
Single tumor > 7 cm and multiple tumors beyond up-to-seven criteria (reassigned BCLC A vs. B)	2.2 (1.6, 3.0)	< 0.001	…	…	2.4 (1.6, 3.5)	< 0.001	…	…
Tumor differentiation (well or moderate vs. poor)	NA	NA	NA	NA	1.5 (1.1, 2.2)	0.02	…	…
MVI (absent vs. present)	NA	NA	NA	NA	2.4 (1.6, 3.5)	< 0.001	1.8 (1.2, 2.7)	0.003

Data in parentheses are 95% confidence intervals

*AFP* alpha-fetoprotein, *BCLC* Barcelona Clinic Liver Cancer, *HBV* hepatitis B virus, *MVI* microvascular invasion, *NA* not applicable, *TTB* total tumor burden, *TTS* total tumor size, *TTV* total tumor volume

#### Patients with complete pathological data

For patients who had complete documentation of tumor differentiation and MVI status (*n* = 385), the multivariable Cox regression analysis showed that TTB remained the most important predictor of ER (HR = 2.0; 95% CI: 1.4, 3.0; *p* < 0.001), with a C-index of 0.610 (95% CI: 0.566, 0.653). Additional factors retained in the final Cox model based on the akaike information criteria included serum AFP level (HR = 1.7; 95% CI: 1.2, 2.5; *p* = 0.007), tumor multiplicity (HR = 1.6; 95% CI: 1.0, 2.5; *p* = 0.06), and MVI (HR = 1.8; 95% CI: 1.2, 2.7; *p* = 0.003) (Table 2).

#### Entire cohort plus cases with inaccurate segmentations

After incorporating 22 cases with inaccurate segmentations into the entire cohort (*n* = 614), TTB remained an independent variable for predicting ER (HR = 1.6; 95% CI: 1.1, 2.3; *p* = 0.009), with a C-index of 0.591 (95% CI: 0.556, 0.626) (Supplementary Material 4 and Table S3).

### Survival analyses

#### TTB and TTS for ER risk stratification

**Fig. 4:**
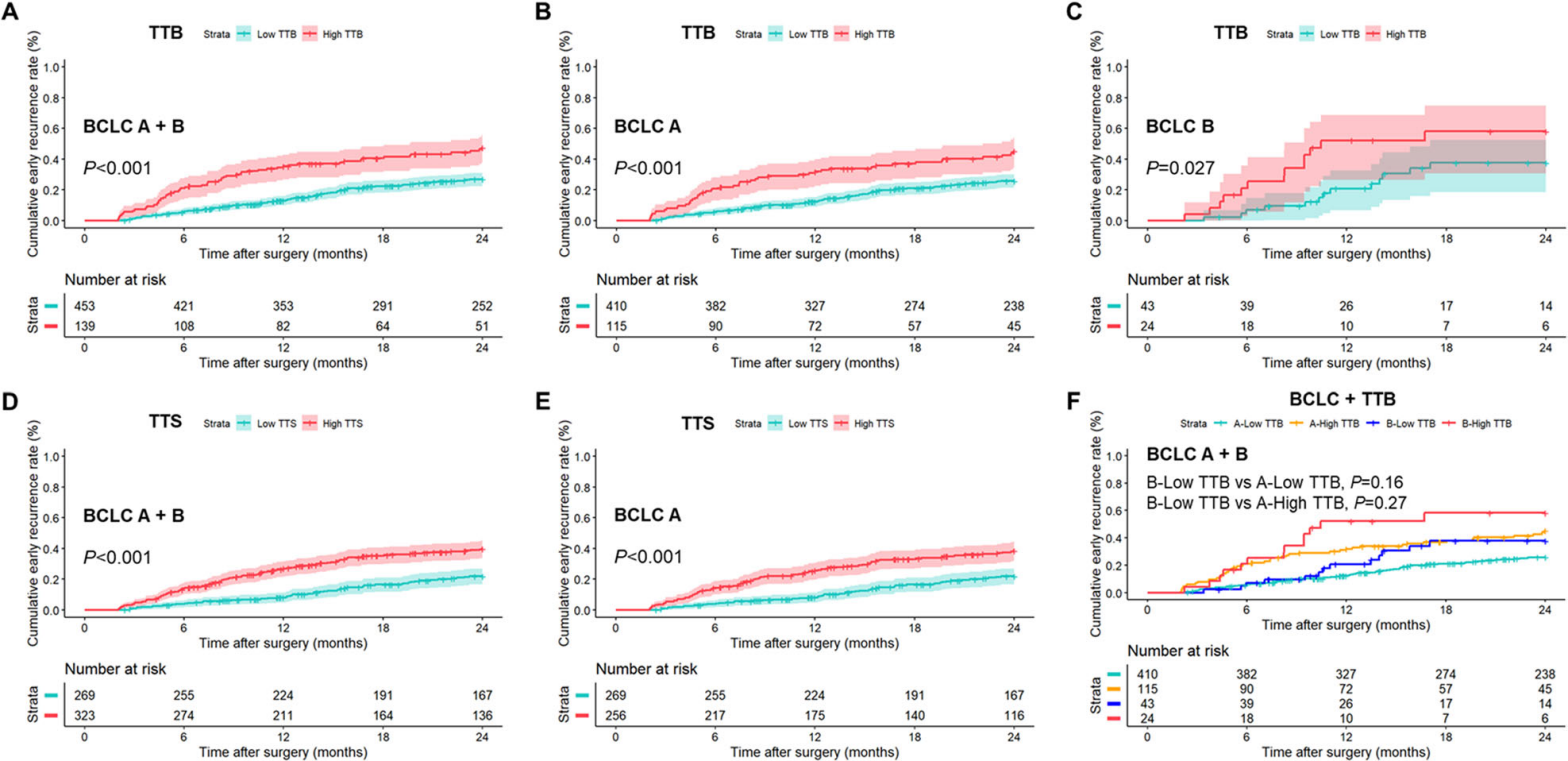
Graphs show cumulative rates of early recurrence between low (< 6.84%) and high (≥ 6.84%) TTB groups in (**A**) all patients, (**B**) patients with BCLC A HCC, and (**C**) patients with BCLC B HCC. Graphs show cumulative rates of early recurrence between low (< 4.1 cm) and high (≥ 4.1 cm) TTS groups in (**D**) all patients and (**E**) patients with BCLC A HCC. **F** Graph shows cumulative rates of early recurrence according to TTB combined with BCLC stage subgroups. BCLC, Barcelona Clinic Liver Cancer; HCC, hepatocellular carcinoma; TTB, total tumor burden; TTS, total tumor size

Using 6.84% as the threshold, TTB stratified all patients into two risk strata for ER (ER rate at 24 months, 26.8% vs. 47.4%; *p* < 0.001), as well as BCLC A patients (ER rate at 24 months, 25.8% vs. 45.1%; *p* < 0.001) and BCLC B patients (ER rate at 24 months, 37.7% vs. 58.1%; *p* = 0.027), respectively (Table 3; Fig. 4A–C). Additionally, TTS gave two risk strata for ER among all patients (ER rate at 24 months, 21.9% vs. 39.9%; *p* < 0.001) and BCLC A patients (ER rate at 24 months, 21.9% vs. 38.6%; *p* < 0.001), respectively (Table 3; Fig. 4D, E). However, all BCLC B patients had high TTS and thus could not be stratified into two risk strata for ER according to TTS (ER rate at 24 months, 45.3%) (Table 3).

**Table 3:** ER rates at 6, 12, 18, and 24 months and hazard ratios according to TTB, TTS, BCLC stage, and modified BCLC stage

Group	No. of patients	ER rate at 6 months, %	ER rate at 12 months, %	ER rate at 18 months, %	ER rate at 24 months, %	Hazard ratio	*p-*value
**All patients**							
TTB						2.3 (1.6, 3.1)	< 0.001
Low	453	6.0 (3.8, 8.2)	13.0 (9.8, 16.1)	22.4 (18.3, 26.3)	26.8 (22.4, 31.0)		
High	139	20.9 (13.8, 27.4)	35.4 (26.8, 43.0)	40.5 (31.5, 48.4)	47.4 (37.8, 55.6)		
TTS						2.2 (1.6, 3.1)	< 0.001
Low	269	4.1 (1.7, 6.5)	8.0 (4.7, 11.2)	16.4 (11.7, 20.9)	21.9 (16.4, 26.9)		
High	323	14.0 (10.1, 17.7)	26.8 (21.8, 31.6)	35.3 (29.6, 40.5)	39.9 (33.9, 45.3)		
BCLC stage						1.8 (1.2, 2.7)	0.007
A	525	9.2 (6.7, 11.6)	16.6 (13.3, 19.7)	24.5 (20.6, 28.2)	30.0 (25.7, 34.0)		
B	67	12.1 (3.9, 19.7)	32.3 (19.5, 43.1)	45.3 (30.2, 57.2)	45.3 (30.2, 57.2)		
Modified BCLC stage						2.8 (1.6, 4.9)	< 0.001
A_n_ (A + B-low TTB)	568	9.0 (6.6, 11.4)	16.8 (13.6, 19.9)	25.3 (21.5, 29.0)	30.5 (26.4, 34.4)		
B_n_ (B-high TTB)	24	21.1 (2.8, 35.9)	52.2 (26.4, 68.9)	58.1 (30.7, 74.7)	58.1 (30.7, 74.7)		
**BCLC A patients**							
TTB						2.2 (1.5, 3.1)	< 0.001
Low	410	5.9 (3.6, 8.1)	12.3 (9.0, 15.4)	21.0 (16.8, 25.0)	25.8 (21.2, 30.2)		
High	115	20.9 (13.1, 28.0)	31.9 (22.7, 40.0)	36.9 (27.2, 45.4)	45.1 (34.5, 54.0)		
TTS						2.1 (1.5, 3.0)	< 0.001
Low	269	4.1 (1.7, 6.5)	8.0 (4.7, 11.2)	16.4 (11.7, 20.9)	21.9 (16.4, 26.9)		
High	256	14.5 (10.0, 18.7)	25.5 (19.9, 30.8)	33.0 (26.8, 38.7)	38.6 (32.0, 44.6)		
**BCLC B patients**							
TTB						2.3 (1.1, 5.0)	0.027
Low	43	7.1 (0.0, 14.5)	20.8 (6.7, 32.7)	37.7 (18.6, 52.3)	37.7 (18.6, 52.3)		
High	24	21.1 (2.8, 35.9)	52.2 (26.4, 68.9)	58.1 (30.7, 74.7)	58.1 (30.7, 74.7)		
TTS						…	…
Low	0	…	…	…	…		
High	67	12.1 (3.9, 19.7)	32.3 (19.5, 43.1)	45.3 (30.2, 57.2)	45.3 (30.2, 57.2)		

Numbers in parentheses are 95% confidence intervals

*BCLC* Barcelona Clinic Liver Cancer, *ER* early recurrence, *TTB* total tumor burden, *TTS* total tumor size

To identify subgroups of BCLC B patients who had favorable prognosis after resection, combinations of BCLC stage and TTB were analyzed. BCLC B low-TTB patients demonstrated comparable risk for ER to that of BCLC A patients with either low (ER rate at 24 months, 37.7% vs. 25.8%; *p* = 0.16) or high (ER rate at 24 months, 37.7% vs. 45.1%; *p* = 0.27) TTB (Table 3; Fig. 4F).

#### Construction of modified BCLC algorithm for stage A and B HCC

**Fig. 5:**
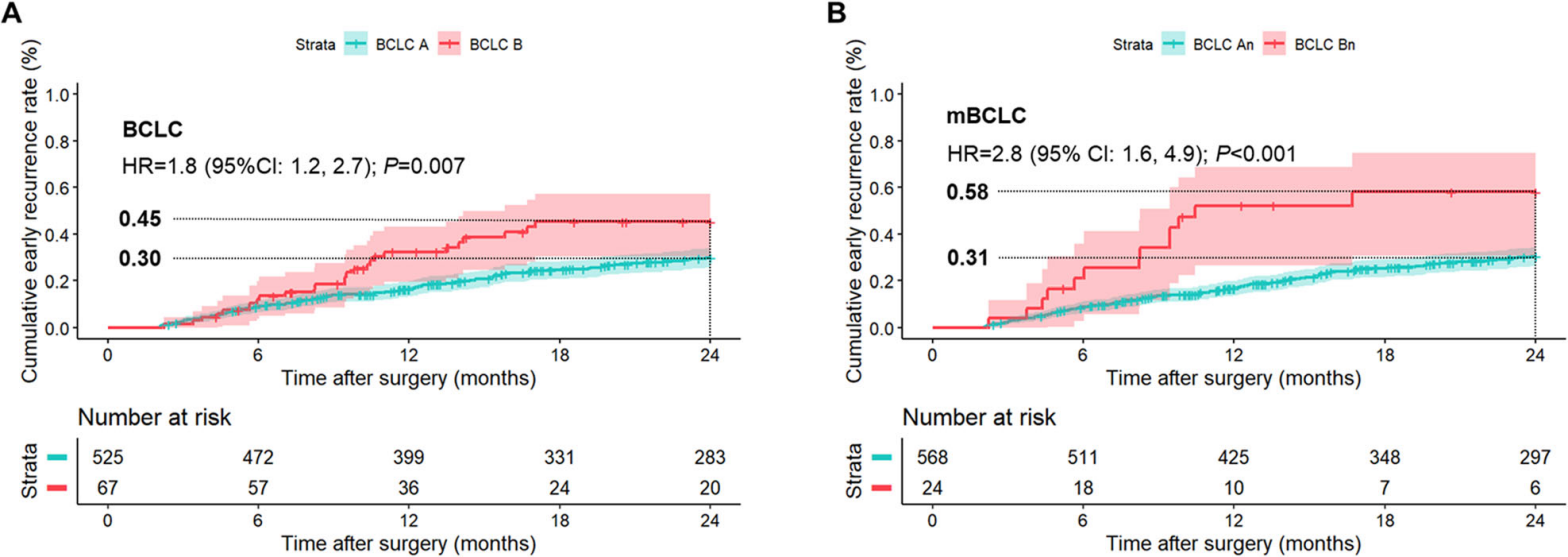
Graphs show cumulative rates of early recurrence according to the (**A**) original and (**B**) modified BCLC algorithms. The modified BCLC algorithm provided a greater separation of the cumulative incidence curves compared with the original system. BCLC, Barcelona Clinic Liver Cancer; CI, confidence interval; HR, hazard ratio; mBCLC, modified Barcelona Clinic Liver Cancer

The ER rate at 24 months was 30.0% (95% CI: 25.7, 34.0) for BCLC A patients vs. 45.3% (95% CI: 30.2, 57.2) for BCLC B patients (HR = 1.8; 95% CI: 1.2, 2.7; *p* = 0.007) (Table 3; Fig. 5A). By integrating TTB into the current BCLC system, a modified BCLC algorithm was constructed: *(a)* BCLC stage A_n_ (*n* = 568), constituting of the original BCLC A patients plus those BCLC B patients with low TTB; and *(b)* BCLC stage B_n_ (*n* = 24), consisting of the remaining BCLC B patients with high TTB. Based on the modified BCLC algorithm, the ER rate at 24 months was 30.5% (95% CI: 26.4, 34.4) for BCLC A_n_ patients vs. 58.1% (95% CI: 30.7, 74.7) for BCLC B_n_ patients (HR = 2.8; 95% CI: 1.6, 4.9; *p* < 0.001) (Table 3; Fig. 5B).

**Fig. 6:**
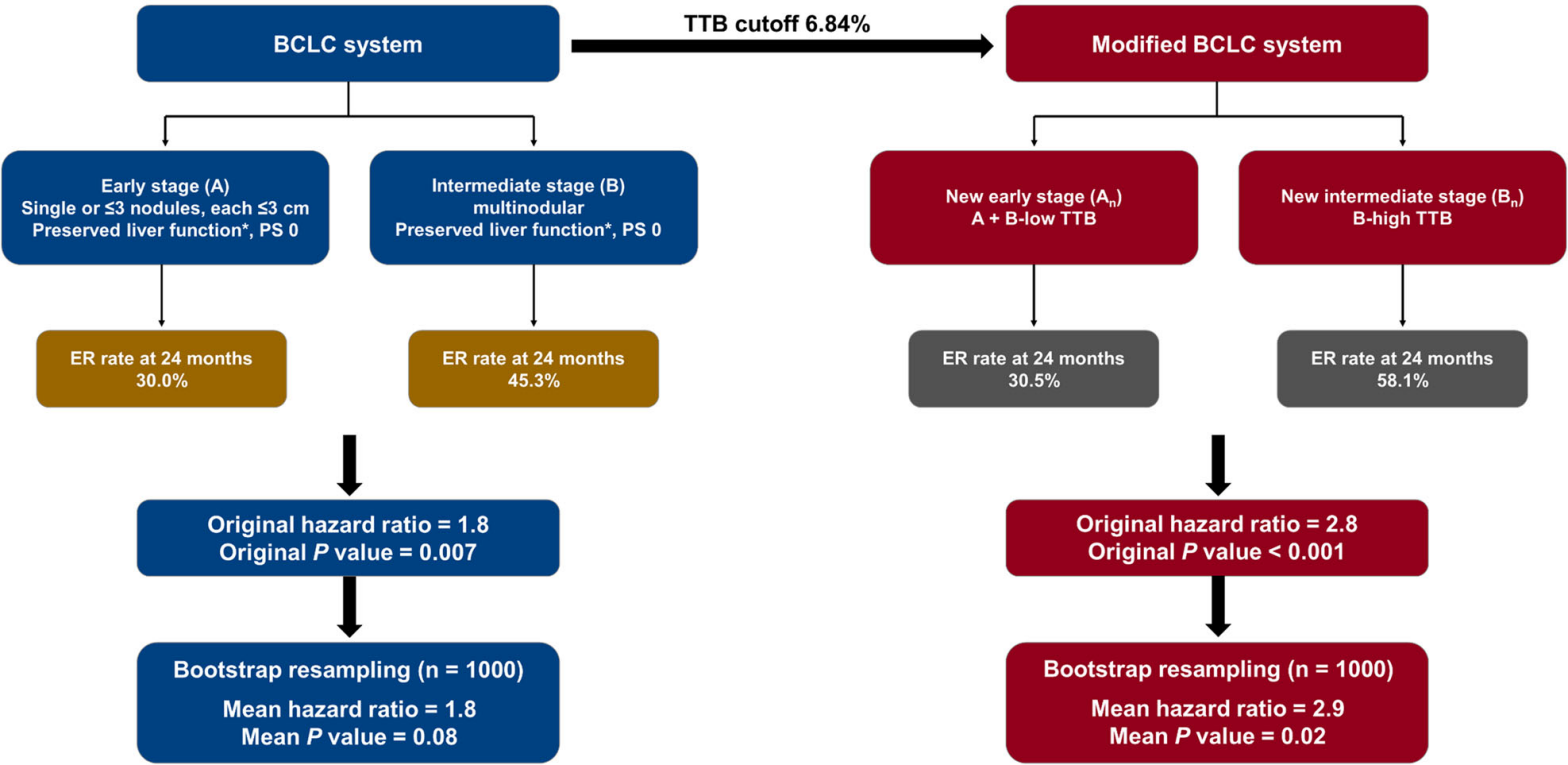
Schematic diagrams of BCLC and modified BCLC systems. The modified BCLC system provided an improved prognostic risk stratification for patients with BCLC stage A and B HCC. It also demonstrated a potentially more robust prognostic impact on early recurrence according to bootstrap resampling analysis. BCLC, Barcelona Clinic Liver Cancer; ER, early recurrence; HCC, hepatocellular carcinoma; PS, performance status; TTB, total tumor burden. *Except for those with tumor burden acceptable for Transplant

After bootstrap resampling, BCLC B patients demonstrated a mean HR of 1.8 (95% CI: 1.0, 2.6) for ER relative to BCLC A patients (mean *p* = 0.08), whilst BCLC B_n_ patients demonstrated a mean HR of 2.9 (95% CI: 1.1, 4.7) for ER relative to BCLC A_n_ patients (mean *p* = 0.02) (Fig. 6).

#### Sensitivity analysis for patients without adjuvant therapy after surgery

For patients who did not receive adjuvant therapies after surgery (*n* = 488), similar results were also obtained for survival analyses based on TTB, TTS, BCLC, and modified BCLC algorithms (Supplementary Material 5, Table S4, Figs. S3 and 4).

Comparisons of patient characteristics between low and high TTB groups are provided in Supplementary Material 6 and Table S5.

## Discussion

This study aimed to evaluate the prognostic impact of MRI-based automated 3D volumetric parameters in predicting ER of BCLC A and B HCC. Our findings demonstrated that TTB was an independent predictor of postoperative ER, even after adjusting for pathological factors. We identified two ER risk groups based on TTB, with high TTB (≥ 6.84%) patients at elevated ER risk. Additionally, BCLC B low-TTB patients had similar ER risk to BCLC A patients, leading to their recategorization in a modified BCLC algorithm. The modified BCLC system resulted in an improved prognostic stratification for BCLC A and B patients.

Conventional one-dimensional tumor measurements, though user-friendly, provide only a basic size estimation and fail to accurately represent the varied growth patterns and irregular shapes of tumors. Conversely, the 3D volumetric quantification offers a more thorough evaluation of the tumor’s extent, characterized by high reproducibility and interobserver agreement [9]. Previous studies have demonstrated that 3D quantitative tumor burden biomarkers outperformed one-dimensional measurements in HCC prognostication [9–12]. Aligning with these studies, our investigation also revealed that TTB, representing the ratio of total tumor volume to liver volume and the remnant liver function reserve, was independently correlated with ER, whilst TTS was not. Additionally, TTB effectively stratified BCLC B patients into two risk strata, whereas TTS did not. These findings highlight the superior capacity of TTB over traditional diameter-based measurements in assessing ER risk of HCC.

To our knowledge, this is the first study to explore the potential utility of automated 3D quantitative tumor burden for refining BCLC A and B HCC subcategorization. In our study, TTB was the dominant predictor of ER, even after adjusting for pathological factors. Using 1000-bootstrap resampling, the TTB-based modified BCLC system remained significantly associated with ER (mean *p* = 0.02), unlike the original BCLC system (mean *p* = 0.08). These findings signify that the modified BCLC system may have a more robust prognostic impact on ER. Additionally, similar findings were obtained after excluding patients who received adjuvant therapies, suggesting the robustness of TTB in ER risk stratification. However, due to the single-institution retrospective study design and the small number of BCLC B patients (*n* = 67), the modified BCLC system requires further validation and refinement on larger-scale populations before it can be implemented in clinical practice, particularly in terms of generalizability and simplicity.

DL-based automated image segmentation represents a pivotal advancement for precise 3D volumetric analysis. Developed from a dataset of 1889 patients across six tertiary hospitals in China, the models exhibit good accuracy in liver and lesion segmentation. The consistency between automated and manual segmentations of HCC observed in our study underscores the potential of automated segmentation for refining clinical decision-making. To address the limitations of traditional U-Net architecture, incorporating NNU-Net—a self-configuring framework that mitigates the complexities of manual parameter tuning—could yield substantial improvements. Leveraging pre-trained foundation models via transfer learning may further enhance segmentation performance. These strategies aim to enhance model capabilities and streamline the integration of DL segmentation into routine practice, providing a more effective tool for managing HCC patients. However, despite commercial certification, the software used in our study is not yet publicly available.

Noteworthily, a small subset of cases (*n* = 22) presented challenges during automated segmentation and were excluded from volumetric analysis. The inaccurate segmentation observed in these cases could be attributed to various factors, e.g., the presence of an obscure tumor margin, inhomogeneous signal intensity within the tumor, and additional signal interference introduced by peritumoral parenchyma. To improve the accuracy of automated segmentation, further refinement of the DL algorithms is needed. Once this advanced technique is validated and improved in more widespread populations, it is expected to become an applicable workflow in routine practice.

This study had several limitations. First, our study used a single-institution retrospective cohort, lacking external validation. Expanding our findings to larger-scale multicenter cohorts is necessary to confirm their reproducibility and generalizability. Second, the limited sample size of BCLC B patients warrants further validation of the prognostic utility of TTB in future studies. Third, we only evaluated patients with surgically resected HCC, so our results may not be applicable to nonsurgical patients. Fourth, despite the automated segmentation, there were two steps involving the radiologist’s intervention—the 3D bounding box adjustment (*n* = 25) and the exclusion of inaccurate segmentations (*n* = 22), potentially overestimating the automated technique’s performance. Finally, future multicenter studies are encouraged to externally validate the TTB threshold and test their applicability in different populations.

In conclusion, based on 592 patients who underwent curative liver resection for early to intermediate-stage HCC, TTB derived by MRI-based automated DL segmentation was a useful imaging biomarker for postoperative ER prediction and enabled optimized BCLC subclassification.

## Supplementary information

Supplementary Materials

## Abbreviations

3D: Three-dimensional
AFP: Alpha-fetoprotein
AI: Artificial intelligence
BCLC: Barcelona Clinic Liver Cancer
CI: Confidence interval
DL: Deep learning
DSC: Dice similarity coefficient
ER: Early recurrence
HBV: Hepatitis B virus
HCC: Hepatocellular carcinoma
HR: Hazard ratio
ICC: Intraclass correlation coefficient
IQR: Interquartile range
MVI: Microvascular invasion
TACE: Transarterial chemoembolization
TLV: Total liver volume
TTB: Total tumor burden
TTS: Total tumor size
TTV: Total tumor volume
