# Supplementary material for: Deep learning-based 3D quantitative total tumor burden predicts early recurrence of BCLC A and B HCC after resection
Source: Eur Radiol. 2024 Jul 19;35(1):127–39. doi: 10.1007/s00330-024-10941-y (PMC11632001; doi:10.1007/s00330-024-10941-y)
Supplement: Supplementary file 1 — Supplementary Materials [file 330_2024_10941_MOESM1_ESM.pdf]

Supplementary Materials

Supplementary Material 1 MRI technique (Page 2)

Supplementary Material 2 One-dimensional measurement (Page 3)

Supplementary Material 3 Deep learning algorithms for automated segmentation and volumetric quantification (Page 4)

Supplementary Material 4 Predictors for early recurrence based on Cox regression analyses (adding 22 cases with inaccurate image segmentations into the entire cohort) (Page 8)

Supplementary Material 5 Sensitivity analysis for patients without adjuvant therapy after surgery (Page 9)

Supplementary Material 6 Comparisons of patient characteristics between low and high TTB groups (Page 10)

Table S1 MRI sequences and parameters (Page 11)

Table S2 Dice similarity coefficients between the automated and manual tumor segmentations (Page 14)

Table S3 Predictors for early recurrence based on Cox regression analyses (adding 22 cases with inaccurate image segmentations into the entire cohort) (Page 15)

Table S4 ER rates at 6, 12, 18, and 24 months and hazard ratios according to TTB, TTS, BCLC stage, and modified BCLC stage in patients without adjuvant therapies after surgery (Page 16)

Table S5 Comparisons of patient characteristics between low and high TTB groups (Page 17)

Figure S1 3D-Unet architectures of liver and tumor segmentation models (Page 18)

Figure S2 Violin plots demonstrating DSCs between automated and manual tumor segmentations (Page 19)

Figure S3 Cumulative incidence of early recurrence curves according to TTB and TTS among patients without adjuvant therapies after surgery (Page 20)

Figure S4 Cumulative incidence of early recurrence curves according to the original and modified BCLC algorithms among patients without adjuvant therapies after surgery (Page 21)

Supplementary Material 1 MRI technique

Hepatobiliary contrast agent-enhanced magnetic resonance imaging (HCA MRI) was performed with four 3.0-T systems (GE SIGNA™ Architect; GE SIGNA™ Premier; GE Discovery MR 750; Siemens MAGNETOM Skyra) and a 1.5-T system (uMR588), and extracellular contrast agent-enhanced MRI (ECA MRI) was performed with five 3.0-T systems (Siemens MAGNETOM Skyra; Siemens TrioTim; GE SIGNA™ Architect; GE Discovery MR 750; Philips Ingenia Elition X) and two 1.5-T systems (Siemens Avanto; uMR588). Liver MRI protocols involved T2-weighted imaging, diffusion-weighted imaging (b values: 0-1200 s/mm^2) with apparent diffusion coefficient (ADC) maps, T1-weighted in- and opposed-phase imaging, and dynamic T1- weighted imaging before and after injection of contrast agent in the late arterial phase, portal venous phase (60 s), delayed phase (ECA MRI; 180 s) or transitional phase (HCA MRI; 180 s), and hepatobiliary phase (HCA MRI; 20 minutes). The arterial phase images were obtained either by the acquisition triggered 7 s after arrival of the contrast bolus in the celiac trunk or a multiple arterial phase (MAP) imaging technique. Specifically, the MAP images were acquired with an 18 s breath hold 20 s after the contrast media injection, and further reconstructed with a temporal resolution of 3 s. For HCA MRI, gadoxetate disodium (Primovist®; Bayer Schering Pharma AG) was administered intravenously at 1.0-2.0 ml/s (0.025 mmol/kg of body weight), with an immediately followed 20-30 ml saline flush. For ECA MRI, gadopentetate dimeglumine (Magnevist®; Bayer Schering Pharma AG) or gadoterate meglumine (Dotarem®; Guerbet) or gadobenate dimeglumine (MultiHance®; Bracco) was administered intravenously at 2.5 ml/s (0.1 mmol/kg of body weight). MRI sequences and parameters are detailed in **Table S1.**

Supplementary Material 2 One-dimensional measurement

One-dimensional measurement of tumors was performed by two abdominal radiologists (H.W. and H.Y.J., with 5 and 8 years of experience in liver MRI, respectively), who were aware that all patients had HCCs but were blinded to other information.

On a per-lesion basis, the following parameters were recorded for all HCC lesions: *(a)* number of tumors, defined as the number of definite intrahepatic HCC lesions according to the version 2018 Liver Imaging Reporting and Data System[1]; *(b)* tumor size (cm), defined as largest outer-edge-to-outer-edge dimension of a tumor; and *(c)* tumor location according to the Couinaud classification.

First, the two readers (H.W. and H.Y.J.) independently assessed the tumor number and location on MR images, with all discrepancies resolved by a third senior abdominal radiologist (R.B.L.) with over 20 years of experience in liver MRI. After reaching the consensus on tumor number and location, the two readers (H.W. and H.Y.J.) independently measured the tumor size. Size of each tumor was finally averaged by the measurements of the two readers.

Total tumor size (TTS) was defined as the sum of size of all HCC lesions, Additionally, the single tumor >7 cm and multiple tumors beyond up-to-seven criteria were assessed to reassign the BCLC staging. Patients with single HCC >2 and ≤7 cm or multiple HCCs within up-to-seven criteria were classified as reassigned BCLC stage A, whilst those with single HCC >7 cm or multiple HCCs beyond up-to-seven criteria were classified as reassigned BCLC stage B[2].

References

1. CT/MRI Liver Imaging Reporting and Data System version 2018. American College of Radiology Web site. https://www.acr.org/-/media/ACR/Files/RADS/LI-RADS/LI-RADS-2018-Core.pdf. Accessed December 1, 2022.

2. Wang YY, Zhong JH, Xu HF et al (2019) A modified staging of early and intermediate hepatocellular carcinoma based on single tumour >7 cm and multiple tumours beyond up-to-seven criteria. Aliment Pharmacol Ther 49:202-210

Supplementary Material 3 Deep learning algorithms for automated segmentation and volumetric quantification

Dataset for the Development of Automated Segmentation Models

A total of 1889 patients with focal liver lesions (FLLs) (i.e., hepatocellular carcinoma, hemangioma, and hepatic cyst) from six tertiary hospitals in China between December 2013 and February 2021 were included for developing the automated deep learning (DL) segmentation models. Patients were split into the training (n=1511), validation (n=189), and test (n=189) sets at the ratio of 8:1:1. All magnetic resonance (MR) images in DICOM format were exported from the picture archiving and communication system. Manual segmentations of FLLs were performed by two abdominal radiologists (both with 5 years of experience in liver MRI), avoiding intrahepatic vasculatures. Each radiologist segmented 944 and 945 patients, respectively. Segmentations were performed for T2-weighted imaging (T2WI), diffusion-weighted imaging (DWI) (b value of 800 s/mm^2), in- and opposed-phase imaging, pre- and post-contrast enhanced T1-weighted imaging (T1WI) during late arterial phase, portal venous phase, delayed phase (for extracellular contrast agent-enhanced MRI), transitional phase and hepatobiliary phase (for hepatobiliary contrast agent-enhanced MRI). To allow for quality control for manual segmentations, all regions of interest (ROIs) were inspected by a senior radiologist with 30 years of experience in liver MRI. For the unqualified segmentations, manual adjustment was performed by the two junior radiologists. The resulting sketched images served as input data to train the automated segmentation models.

Automated Segmentation Model Training, Validation and Test

The automated segmentation models were trained using a sequential modular approach. First of all, a three-dimensional convolutional neural network (3D-CNN) model [1] was utilized to generate a liver segmentation mask. During this step, the liver region was delineated from the surrounding abdominal organs on MR images to facilitate a focused analysis on the liver while avoiding the interference from adjacent organs. This algorithm utilized an encoder-decoder architecture with 3D convolutions

and pooling layers, complemented by the Rectified Linear Unit (RLU) activation and batch normalization. Skip connections were established between corresponding layers of the encoder and decoder. The output layer consisted of two branches, which were used for liver boundary segmentation and pixel-level liver region segmentation, respectively. After obtaining accurate liver segmentation masks and delineating intricate liver anatomical strictures, rigorous image registration was implemented by aligning multiple MRI sequences with a standardized spatial reference framework. This crucial step enhanced the spatial coherence between the liver segmentation masks across various MRI sequences.

To enhance the accuracy of FLL detection, segmented liver images were converted into input data for lesion detection algorithm. Thereafter, an advanced deep learning algorithm was created for the automated detection of FLLs in each sequence of contrast-enhanced MR images. The core algorithm is the use of a 3D-CNN model known as the Unified Multi-Sequence Lesion Detector (MSLD), which incorporated two primary elements: *(a)* a series of Single Lesion Detectors (SLD) used for independent lesion detection in each sequence, and *(b)* a False Positive Reduction (FPR) module designed to mitigate false alarms in the identified lesions. By using the MSLD model, each detected lesion was annotated by a bounding box in each sequence.

To handle the diversity of MRI sequences, we developed Single Lesion Detectors (SLDs) customized for each sequence, extending the Mask region-based convolutional neural network (R-CNN) [2] framework to process 3D input images. Four SLDs sharing the same architecture effectively accommodated the variations in tissue appearances across various sequence groups, including pre-contrast T1WI, post-contrast T1WI, T2WI, and DWI. The SLD framework included the Region Proposal Network (RPN), ROI alignment, lesion identification, and segmentation modules. Notably, the introduction of an adaptive receptive field allowed for the extraction of global features within the slices. Additionally, the Feature Pyramid Network (FPN) [3] captured multi-scale information to ensure robust perceptual capabilities. The training process included the normalization of preprocessed images (with a spacing of 2×2×2

mm³), the cropping of these images into 160×160×160 patches, and the utilization of the Adam optimizer for a total of 200 epochs with a batch size set at eight. The initial learning rate was 0.001, decaying every 50 epochs.

Multiple sequences were used to diminish the influence of image artifacts on lesion detection. In automated FLL detection within the SLD section, bounding boxes for various sequences were cross-referenced to identify candidate lesions. To diminish false alarms stemming from artifacts, a dedicated FPR module integrated a 3D-CNN for feature extraction from each ROI, followed by feature integration from multiple sequences for binary predictions. Standardizing ROI dimensions to 32×32×32 ensured uniformity for typical lesion sizes. Model training spanned 200 epochs with an initial learning rate of 0.001, decayed by 0.1 every 30 epochs, and a batch size of 64 for optimized training.

Lesion segmentation was accomplished by employing a 3D-UNet framework, characterized by an encoder-decoder architecture that incorporated 3D convolutions and pooling layers. This framework was reinformed with Rectified Linear Unit (RLU) activation and batch normalization. The encoder and decoder were interconnected, each consisting of 4 layers of 3D conv-bn-RLU. After the final decoder layer, a 3D conv-bn-RLU layer was integrated for the ultimate lesion segmentation prediction. The model employed the Adam optimizer with an initial learning rate of 0.001, gradually reduced by a factor of 0.1 every 30 epochs, culminating after 60 training epochs.

Automated Segmentation Model Performance

The accuracy of the automated liver and lesion segmentation models was verified on the **test set.** The mean Dice similarity coefficient (DSC) between automated and manual liver segmentation was 0.95±0.11 (range: 0.79-0.99) for all sequences. A successful segmentation was determined by a DSC>0.9. Accordingly, the automated liver segmentation achieved a success rate of 92% (174/189) on the test set. Moreover, the mean DSC between automated and manual lesion segmentations was 0.78±0.16 (range: 0.59-0.96) for all sequences.

3D Volumetric Quantification Analysis

For 3D volumetric quantification analysis, a DL-based automated segmentation software was utilized to measure the liver volume (cm^3) and tumor volume (cm^3). The liver volume was calculated by multiplying the number of pixels within the DL-generated liver segmentation by the physical spacing in the z, y, and x directions. Similarly, the volume of each tumor was calculated by multiplying the number of pixels within the DL-generated lesion segmentation by the corresponding physical spacing in the z, y, and x directions.

References

1. Han X, Wu X, Wang S, Xu L, Xu H, Zheng D, et al. Automated segmentation of liver segment on portal venous phase MR images using a 3D convolutional neural network. Insights Imaging. 2022 Feb;13(1):26.
2. He K, Gkioxari G, Dollár P, Girshick R. Mask R-CNN, in 2017 IEEE International Conference on Computer Vision (ICCV). 2017. p. 2980-2988.
3. Lin TY, Doll´ar P, Girshick R, He K, Hariharan B, and Belongie S. Feature Pyramid Networks for Object Detection, in 2017 IEEE Conference on Computer Vision and Pattern Recognition (CVPR). 2017. p. 936-944.

Supplementary Material 4 Predictors for early recurrence based on Cox regression analyses (adding 22 cases with inaccurate image segmentations into the entire cohort)

All Patients. — For all patients (n=614), 8 parameters, including serum AFP level, postoperative adjuvant therapy, BCLC stage, tumor multiplicity, TTS, TTV, TTB, and the single tumor >7 cm and multiple tumors beyond up-to-seven criteria, were significantly associated with early recurrence (ER) at univariable Cox regression analyses (*P*<0.05 for all). On subsequent multivariate Cox regression analysis, serum AFP level (hazard ratio [HR]=1.4; 95% CI: 1.0, 1.9; *P*=0.05), postoperative adjuvant therapy (HR=1.3; 95%CI: 0.9, 1.9; *P*=0.10), TTS (HR=1.7; 95%CI: 1.2, 2.5; *P*=0.003) and TTB (HR=1.6; 95%CI: 1.1, 2.3; *P*=0.009) were predictors retained in the final Cox model based on the Akaike Information Criteria (**Table S3**). The C-index for TTB in predicting the risk of ER was 0.591 (95%CI: 0.556, 0.626).

Patients with Complete Pathological Data. — For patients who had complete documentation of tumor differentiation and MVI status (n=394), the multivariable Cox regression analysis showed that age (HR=0.6; 95%CI: 0.4, 1.0; *P*=0.06), TTS (HR=1.7; 95%CI: 1.1, 2.7; *P*=0.03), TTB (HR=1.6; 95%CI: 1.0, 2.4; *P*=0.03), and MVI (HR=1.9; 95%CI: 1.3, 2.8; *P*=0.001) were predictors retained in the final Cox model based on the Akaike Information Criteria (**Table S3**). The C-index for TTB in predicting the risk of ER was 0.602 (95%CI: 0.559, 0.644).

Supplementary Material 5 Sensitivity analysis for patients without adjuvant therapy after surgery

For patients who did not receive adjuvant therapies after surgery (n=488), TTB also gave two prognostically distinct risk strata for ER in all (*P*<0.001), BCLC A (*P*<0.001), and BCLC B (*P*=0.014) patients, respectively (**Table S4 and Fig. S3A-C**). Additionally, TTS gave two risk strata for ER among all patients (ER rate at 24 months, 20.3% vs 38.4%; P<0.001) and BCLC A patients (ER rate at 24 months, 20.3% vs 37.3%; P<0.001), respectively (**Table S4; Fig. S3D, E**). However, all BCLC B patients had high TTS and thus could not be stratified into two risk strata for ER according to TTS (ER rate at 24 months, 42.5%) (**Table S4**). Compared to the original BCLC system (ER rate at 24 months: 28.0% vs 42.5% for stage A vs B; HR=1.9; 95%CI: 1.2, 3.1; *P*=0.008), the modified BCLC algorithm led to an ER rate at 24 months of 28.3% for stage A_n and 60.5% for stage B_n (HR=3.3; 95%CI: 1.8, 6.2; *P*<0.001) (**Table S4 and Fig. S4**).

After bootstrap resampling, BCLC B patients demonstrated a mean HR of 2.0 (95%CI: 1.0, 3.0) for ER relative to BCLC A patients (mean *P*=0.09), whilst BCLC B_n patients demonstrated a mean HR of 3.5 (95%CI: 1.1, 6.0) for ER relative to BCLC A_n patients (mean *P*=0.02).

Supplementary Material 6 Comparisons of patient characteristics between low and high TTB groups

In terms of clinical-radiological characteristics, patients with high TTB had less frequent liver cirrhosis (30.9% [43/139] vs 58.5% [265/453]; *P*<0.001) than those with low TTB, with more frequent AFP level ≥400 ng/mL (30.9% [43/139] vs 21.4% [97/453]; *P*=0.02), more advanced BCLC stage (BCLC B: 17.3% [24/139] vs 9.5% [43/453]; *P*=0.01), more frequent use of postoperative adjuvant therapies (24.5% [34/139] vs 15.5% [70/453]; *P*=0.02), and larger TTS (median, 8.1 vs 3.7 cm; *P*<0.001). Regrading pathological characteristic, the presence of MVI was more frequently found in patients with high TTB (71.8% [74/103] vs 38.8% [111/286]; *P*<0.001) than in those with low TTB (**Table S5**).

Table S1 MRI sequences and parameters

Sequence	T1-weighted IP and OP imaging	Dynamic T1-weighted 3D GRE	T2-weighted 2D FSE	Diffusion-weighted imaging†
**GE Discovery MR 750 3.0 Tesla (16-channel phased-array torsor coil)**				
Repetition time (ms)	150	4.1	6315	9230
Echo time (ms)	2.5/1.3	1.9	78	Minimum
Flip angle (°)	70	15	111	90
Section thickness (mm)	6	2	6	6
Spacing (mm)	2	-	2	2
Matrix size	288×192	512×512	288×244	128×128
Field of view (mm^2)	420×420	380×300	360×280	360×380
Acquisition time (s)	31	15	RG	RG
Fat suppression	No	Yes	Yes	Yes
**GE SIGNA™ Architect 3.0 Tesla (30-channel body anterior coil)**				
Repetition time (ms)	233.8	3.9	2400	5000
Echo time (ms)	2.3/1.1	1.7	85	Minimum
Flip angle (°)	55	15	111	90
Section thickness (mm)	7	3	7	7
Spacing (mm)	2	-	2	2
Matrix size	160×288	320×240	320×192	160×128
Field of view (mm^2)	380×323	380×380	380×304	380×342
Acquisition time (s)	18	15	34	RG
Fat suppression	No	Yes	Yes	Yes
**GE SIGNA™ Premier 3.0 Tesla (30-channel body anterior coil)**				
Repetition time (ms)	146.8	3.2	2200	5000
Echo time (ms)	2.3/1.1	1.4	85	Minimum
Flip angle (°)	55	15	111	90
Section thickness (mm)	7	2.4	7	7
Spacing (mm)	2	-	2	2
Matrix size	320×192	320×240	320×224	120×240
Field of view (mm^2)	342×380	380×380	304×380	380×380
Acquisition time (s)	16	15	47	RG
Fat suppression	No	Yes	Yes	Yes
**Siemens MAGNETOM Skyra 3.0 Tesla (18-channel body array coil)**				
Repetition time (ms)	81	3.95	2160	5600
Echo time (ms)	2.72/1.4	1.92	100	68
Flip angle (°)	70	9	160	90
Section thickness (mm)	6	2.5	6	6
Spacing (mm)	1.8	-	1.8	1.8
Matrix size	352×286	352×256	320×288	100×76
Field of view (mm^2)	400×325	400×296	433×433	380×289
Acquisition time (s)	24	14	36	233
Fat suppression	No	Yes	Yes	Yes

**Siemens TrioTim 3.0 Tesla (8-channel body anterior coil)**				
Repetition time (ms)	181	3.47	2700	5900
Echo time (ms)	2.2/3.67	1.25	95	76
Flip angle (°)	65	9	140	90
Section thickness (mm)	6	2.4	6	6
Spacing (mm)	7.8	-	7.8	7.8
Matrix size	256×131	320×133	320×147	192×154
Field of view (mm^2)	410×269	434×257	442×254	393×393
Acquisition time (s)	18	17	RG	245
Fat suppression	No	Yes	Yes	Yes
**Siemens Avanto 1.5 Tesla (30-channel body anterior coil)**				
Repetition time (ms)	72	5.41	2530	3600
Echo time (ms)	4.92/2.22	2.39	84	88
Flip angle (°)	70	10	150	90
Section thickness (mm)	6	2.5	6	6
Spacing (mm)	7.8	-	7.8	7.8
Matrix size	256×158	320×138	256×187	192×115
Field of view (mm^2)	328×225	382×238	293×251	310×232
Acquisition time (s)	16	15	47	92
Fat suppression	No	Yes	Yes	Yes
**Siemens Avanto 1.5 Tesla (8-channel body anterior coil)**				
Repetition time (ms)	87	5.4	2710	2000
Echo time (ms)	4.92/2.22	2.38	84	72
Flip angle (°)	70	10	150	90
Section thickness (mm)	7.5	2	7.5	7.5
Spacing (mm)	9.75	-	9.75	9.75
Matrix size	256×187	320×131	256×177	192×125
Field of view (mm^2)	308×380	241×407	308×380	308×379
Acquisition time (s)	33	15	27	20
Fat suppression	No	Yes	Yes	Yes
**Philips Ingenia Elition X 3.0 Tesla (16-channel body anterior coil)**				
Repetition time (ms)	164.53	4.20	1883.51	1653.65
Echo time (ms)	2.30/1.15	0.00	90	60.29
Flip angle (°)	50	10	90	90
Section thickness (mm)	6	3	6.8	7
Spacing (mm)	7.5	1.5	8.5	8.5
Matrix size	256×201	344×252	272×78	142×140
Field of view (mm^2)	360×360	380×380	380×380	380×380
Acquisition time (s)	11	13	46	52
Fat suppression	No	Yes	Yes	Yes
**uMR588 1.5 Tesla (6-channel body anterior coil)**				
Repetition time (ms)	117.6	4.2	2600	3350
Echo time (ms)	4.7/2.27	1.88	99.2	77
Flip angle (°)	60	10	90	90

Section thickness (mm)	6.5	2.5	6.5	6.5
Spacing (mm)	1.3	-	1.5	10
Matrix size	256×174	256×154	256×168	128×92
Field of view (mm^2)	320×400	255×400	427×320	320×400
Acquisition time (s)	29	13	39	RG
Fat suppression	No	Yes	Yes	Yes

FSE, fast spin-echo; GRE, gradient recall echo; IP, in-phase; MRI, magnetic resonance imaging; NA, not available; OP, opposed-phase; RG, respiratory gating; 3D, three-dimensional; 2D, two-dimensional.

†Images were acquired under free breath.

Table S2 Dice similarity coefficients between the automated and manual tumor segmentations in 40 HCCs among 35 patients

Sequence	Dice similarity coefficients		
	Mean ± SD	Median (IQR)	Range
T2WI	0.87 ± 0.11	0.91 (0.86-0.94)	0.50-0.96
DWI (high b values)	0.86 ± 0.11	0.90 (0.82-0.94)	0.48-0.97
In-phase	0.84 ± 0.10	0.88 (0.79-0.92)	0.52-0.94
PP	0.86 ± 0.09	0.89 (0.83-0.93)	0.55-0.96
AP	0.85 ± 0.10	0.88 (0.84-0.91)	0.48-0.94
PVP	0.83 ± 0.10	0.87 (0.80-0.89)	0.46-0.94
DP (ECA MRI)	0.84 ± 0.08	0.86 (0.81-0.90)	0.63-0.92
TP (HCA MRI)	0.79 ± 0.20	0.85 (0.71-0.94)	0.17-0.94
HBP (HCA MRI)	0.87 ± 0.12	0.90 (0.85-0.94)	0.45-0.95
All	0.85 ± 0.11	0.88 (0.82-0.92)	0.17-0.97

AP, arterial phase; DP, delayed phase; DWI, diffusion weighted imaging; ECA, extracellular contrast agent; HCA, hepatobiliary contrast agent; HBP, hepatobiliary phase; IQR, interquartile range; MRI, magnetic resonance imaging; PP, precontrast phase; PVP, portal venous phase; SD, standard deviation; TP, transitional phase; T2WI, T2-weighted imaging.

Table S3 Predictors for early recurrence based on Cox regression analyses (adding 22 cases with inaccurate image segmentations into the entire cohort)

Variable	All patients (n=614)				Patients with available pathological data (n=394)			
	Univariable analysis		Multivariable analysis		Univariable analysis		Multivariable analysis	
	Hazard ratio	*P* value	Hazard ratio	*P* value	Hazard ratio	*P* value	Hazard ratio	*P* value
Age, y (<65 vs ≥65)	0.7 (0.5, 1.1)	0.13	…	…	0.7 (0.4, 1.0)	0.07	0.6 (0.4, 1.0)	0.06
Sex (female vs male)	1.0 (0.7, 1.6)	0.98	…	…	0.9 (0.5, 1.5)	0.74	…	…
Underlying liver disease (HBV vs non-HBV)	1.0 (0.4, 2.5)	0.95	…	…	0.9 (0.3, 2.5)	0.86	…	…
Cirrhosis (absent vs present)	1.0 (0.8, 1.4)	0.92	…	…	0.9 (0.7, 1.3)	0.63	…	…
Child-Pugh class (A vs B)	1.7 (0.6, 5.5)	0.34	…	…	1.4 (0.4, 5.8)	0.62	…	…
AFP, ng/mL (<400 vs ≥400)	1.5 (1.1, 2.0)	0.01	1.4 (1.0, 1.9)	0.05	2.0 (1.4, 2.9)	<0.001	…	…
Postoperative adjuvant therapy (absent vs present)	1.5 (1.1, 2.2)	0.02	1.3 (0.9, 1.9)	0.10	1.4 (1.0, 2.1)	0.08	…	…
BCLC stage (A vs B)	1.5 (1.0, 2.3)	0.045	…	…	1.5 (0.9, 2.5)	0.14	…	…
Tumor multiplicity (unifocal vs multifocal)	1.7 (1.2, 2.5)	0.004	…	…	1.6 (1.0, 2.5)	0.047	…	…
TTS, cm (<4.1 vs ≥4.1)	2.3 (1.7, 3.1)	<0.001	1.7 (1.2, 2.5)	0.003	2.4 (1.6, 3.6)	<0.001	1.7 (1.1, 2.7)	0.03
TTV, cm^3 (<85.09 vs ≥85.09)	2.2 (1.6, 3.0)	<0.001	…	…	2.4 (1.6, 3.3)	<0.001	…	…
TTB, % (<6.84 vs ≥6.84)	2.3 (1.7, 3.1)	<0.001	1.6 (1.1, 2.3)	0.009	2.4 (1.7, 3.4)	<0.001	1.6 (1.0, 2.4)	0.03
Single tumor >7 cm and multiple tumors beyond up-to-seven criteria (reassigned BCLC A vs B)	2.2 (1.6, 3.0)	<0.001	…	…	2.3 (1.6, 3.3)	<0.001	…	…
Tumor differentiation (well or moderate vs poor)	NA	NA	NA	NA	1.5 (1.1, 2.2)	0.02	…	…
MVI (absent vs present)	NA	NA	NA	NA	2.4 (1.7, 3.5)	<0.001	1.9 (1.3, 2.8)	0.001

Data in parentheses are 95% confidence intervals. AFP, alpha-fetoprotein; BCLC, Barcelona Clinic Liver Cancer; HBV, hepatitis B virus; MVI, microvascular invasion; NA, not applicable; TBS, tumor burden score; TTB, total tumor burden; TTS, total tumor size; TTV, total tumor volume.

Table S4 ER rates at 6, 12, 18, and 24 months and hazard ratios according to TTB, TTS, BCLC stage, and modified BCLC stage in patients without adjuvant therapies after surgery

Group	No. of patients	ER rate at 6 months, %	ER rate at 12 months, %	ER rate at 18 months, %	ER rate at 24 months, %	Hazard ratio	*P* value
**All patients**							
**TTB**						2.4 (1.7, 3.5)	<0.001
Low TTB	383	6.0 (3.6, 8.4)	12.1 (8.7, 15.4)	20.4 (16.1, 24.5)	24.5 (19.9, 29.0)		
High TTB	105	21.0 (12.8, 28.4)	33.9 (24.1, 42.4)	39.2 (28.9, 48.1)	47.5 (36.5, 56.7)		
**TTS**						2.3 (1.6, 3.3)	<0.001
Low TTS	238	4.2 (1.6, 6.8)	7.3 (3.9, 10.6)	15.8 (10.8, 20.4)	20.3 (14.7, 25.5)		
High TTS	250	14.0 (9.6, 18.2)	25.9 (20.2, 31.2)	32.8 (26.5, 38.5)	38.4 (31.7, 44.4)		
**BCLC stage**						1.9 (1.2, 3.1)	0.008
A	438	8.5 (5.8, 11.1)	14.7 (11.2, 17.9)	22.5 (18.4, 26.5)	28.0 (23.5, 32.3)		
B	50	16.4 (5.3, 26.1)	37.0 (21.2, 49.6)	42.5 (25.6, 55.5)	42.5 (25.6, 55.5)		
**Modified BCLC stage**						3.3 (1.8, 6.2)	<0.001
A_n (A + B-Low TTB)	469	8.6 (6.0, 11.1)	15.1 (11.7, 18.3)	23.0 (19.0, 26.9)	28.3 (23.8, 32.4)		
B_n (B-High TTB)	19	26.7 (3.6, 44.3)	60.5 (30.0, 77.7)	60.5 (30.0, 77.7)	60.5 (30.0, 77.7)		
**BCLC A patients**							
**TTB**						2.2 (1.5, 3.3)	<0.001
Low TTB	352	5.7 (3.3, 8.1)	11.3 (7.9, 14.6)	19.6 (15.2, 23.7)	24.0 (19.2, 28.5)		
High TTB	86	19.8 (10.9, 27.8)	28.2 (17.9, 37.1)	34.7 (23.5, 44.1)	44.8 (32.5, 54.8)		
**TTS**						2.2 (1.5, 3.2)	<0.001
Low TTS	238	4.2 (1.6, 6.8)	7.3 (3.9, 10.6)	15.8 (10.8, 20.4)	20.3 (14.7, 25.5)		
High TTS	200	13.5 (8.6, 18.1)	23.4 (17.3, 29.1)	30.6 (23.7, 36.8)	37.3 (29.9, 43.9)		
**BCLC B patients**							
**TTB**						3.0 (1.2, 7.4)	0.014
Low TTB	31	9.9 (0.0, 19.9)	21.9 (4.5, 36.1)	31.4 (10.2, 47.6)	31.4 (10.2, 47.6)		
High TTB	19	26.7 (3.6, 44.3)	60.5 (30.0, 77.7)	60.5 (30.0, 77.7)	60.5 (20.0, 77.7)		
**TTS**						…	…
Low TTS	0	…	…	…	…		
High TTS	50	16.4 (5.3, 26.1)	37.0 (21.2, 49.6)	42.5 (25.6, 55.5)	42.5 (25.6, 55.5)		

Numbers in parentheses are 95% confidence intervals. BCLC, Barcelona Clinic Liver Cancer; ER, early recurrence; TTB, total tumor burden.

Table S5 Comparisons of patient characteristics between low and high TTB groups

Characteristic	Low TTB group (n = 453)	High TTB group (n = 139)	*P* value*
Age, y†	53 (46-61)	55 (45-64)	0.73
Sex			0.45
Female	60 (13.2)	15 (10.8)	
Male	393 (86.8)	124 (89.2)	
Underlying liver disease			0.60
HBV	437 (96.5)	136 (97.8)	
Other	16 (3.5)	3 (2.2)	
Cirrhosis	265 (58.5)	43 (30.9)	<0.001
Child-Pugh class			0.90
A	447 (98.7)	138 (99.3)	
B	6 (1.3)	1 (0.7)	
AFP, ng/mL			0.02
<400	356 (78.6)	96 (69.1)	
≥400	97 (21.4)	43 (30.9)	
BCLC stage			0.01
A	410 (90.5)	115 (82.7)	
B	43 (9.5)	24 (17.3)	
Postoperative adjuvant therapy	70 (15.5)	34 (24.5)	0.02
Contrast agent type of MRI			0.15
ECA	313 (69.1)	87 (62.6)	
HCA	140 (30.0)	52 (37.4)	
Total liver volume, cm^3†	1211.39 (1062.76-1377.89)	1409.99 (1178.78-1597.78)	<0.001
Tumor characteristics			
Tumor multiplicity			0.33
Unifocal	390 (86.1)	115 (82.7)	
Multifocal	63 (13.9)	24 (17.3)	
TTS, cm†	3.7 (2.9-4.9)	8.1 (7.3-10.4)	<0.001
TTV, cm^3†	13.71 (6.50-29.06)	184.39 (127.47-324.58)	<0.001
TTB, %†	1.12 (0.55-2.54)	13.20 (10.31-21.01)	<0.001
Tumor differentiation§			0.17
Well or Moderate	311 (69.3)	87 (63.0)	
Poor	138 (30.7)	51 (37.0)	
MVI§			<0.001
Absent	175 (61.2)	29 (28.2)	
Present	111 (38.8)	74 (71.8)	

Unless indicated otherwise, data are the number of patients, with percentages in parentheses. AFP, alpha-fetoprotein; BCLC, Barcelona Clinic Liver Cancer; ECA, extracellular contrast agent; HBV, hepatitis B virus; HCA, hepatobiliary contrast agent; MRI, magnetic resonance imaging; MVI, microvascular invasion; TTB, total tumor burden; TTS, total tumor size; TTV, total tumor volume.

*Differences were compared between the low and high TTB groups by using the Mann-Whitney *U* test for continuous variables, and Chi-squared test or Fisher's exact test for categorical variables, when applicable.

†Data are medians, with interquartile range in parentheses.

§There were 5 and 203 missing values for tumor differentiation and MVI in the entire study cohort, respectively.

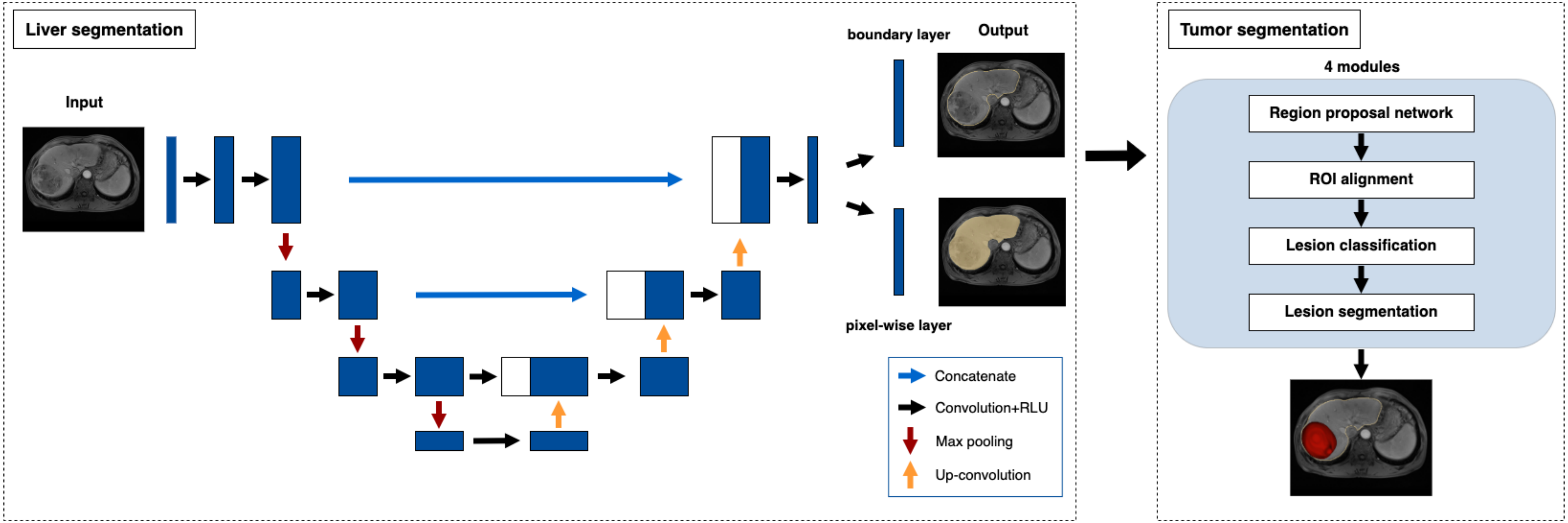

Figure S1 3D-Unet architectures of liver and tumor segmentation models. RLU, rectified linear unit; ROI, region of interest.

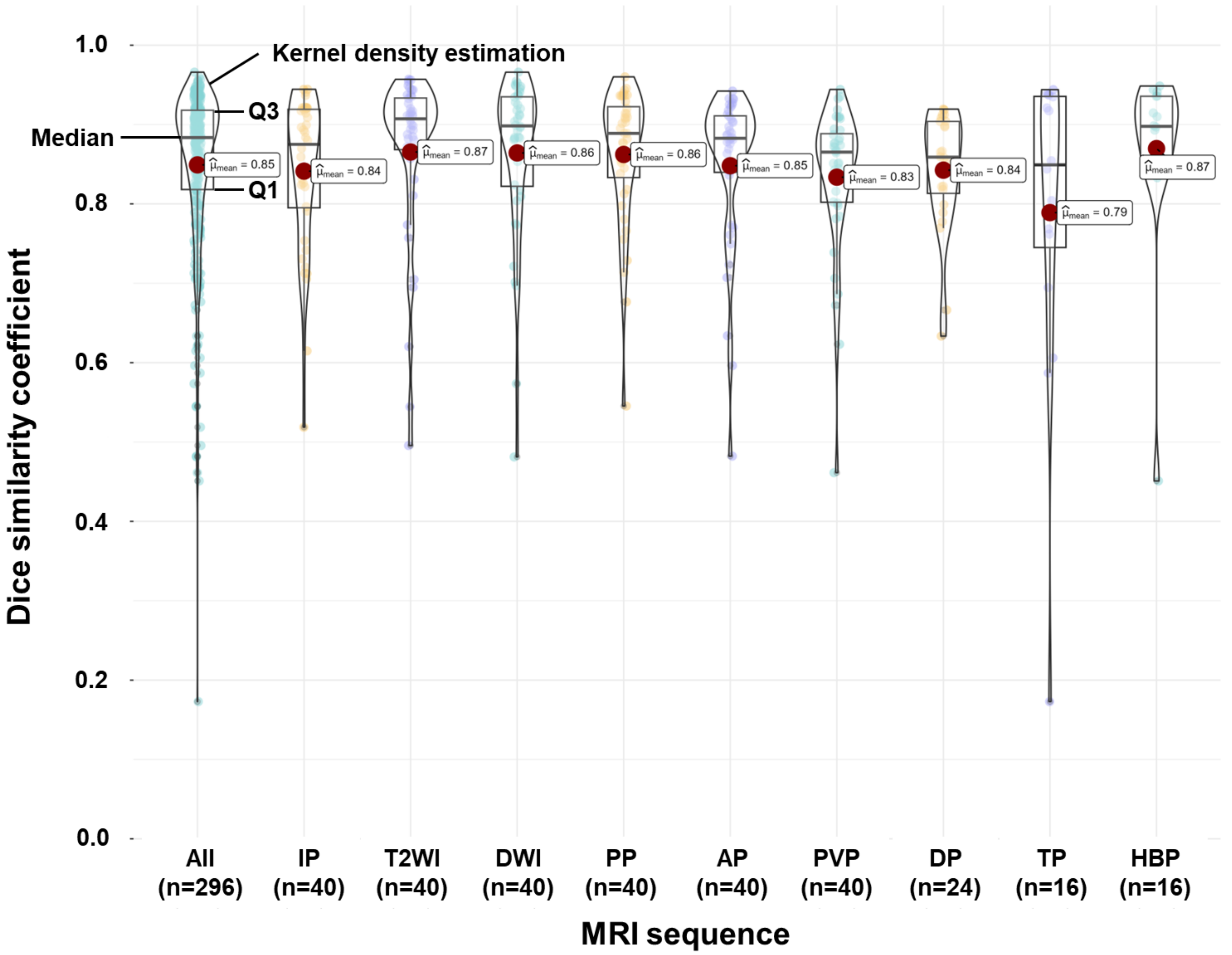

Figure S2 Violin plots demonstrating DSCs between automated and manual tumor segmentations for various MRI sequences in 40 HCCs among 35 patients. The red point at the center of each violin plot indicates the mean value. The box plot shows the median and IQR, with the tails on either side depicting the data beyond the IQR. The width of each violin plot represents the probability density of the DSCs at different values, with wider regions indicating a higher probability density of data points. AP, arterial phase; DP, delayed phase; DSC, Dice similarity coefficient; DWI, diffusion weighted imaging; HBP, hepatobiliary phase; HCC, hepatocellular carcinoma; IP, in-phase; IQR, interquartile range; MRI, magnetic resonance imaging; PP, precontrast phase; PVP, portal venous phase; TP, transitional phase; T2WI, T2-weighted imaging.

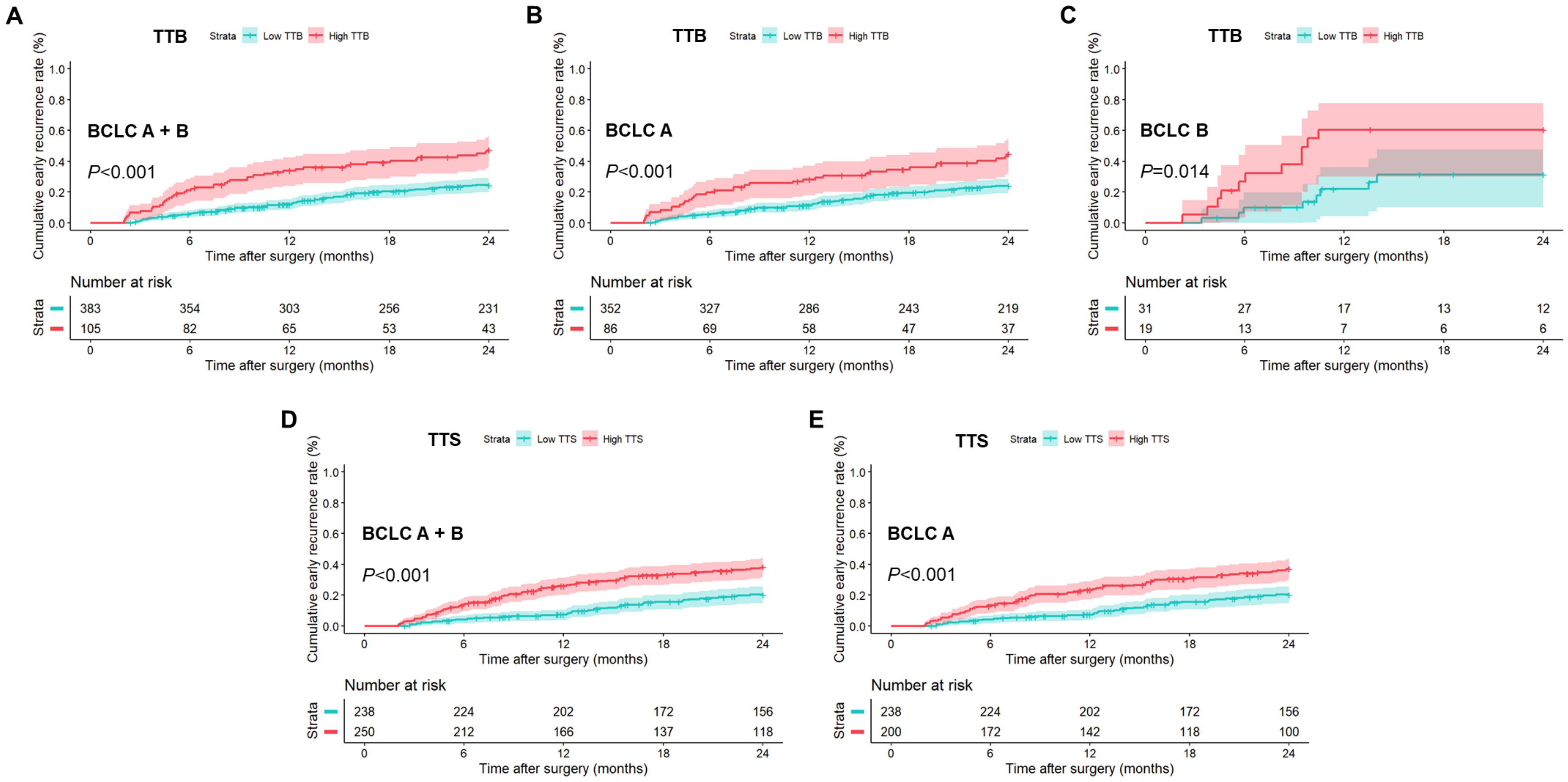

Figure S3 Among patients without adjuvant therapies after surgery, graphs show cumulative rates of early recurrence between low (<6.84%) and high (≥6.84%) TTB groups in (**A**) all patients, (**B**) patients with BCLC A HCC, and (**C**) patients with BCLC B HCC. Graphs show cumulative rates of early recurrence between low (<4.1 cm) and high (≥4.1 cm) TTS groups in (**D**) all patients and (**E**) patients with BCLC A HCC. BCLC, Barcelona Clinic Liver Cancer; HCC, hepatocellular carcinoma; TTB, total tumor burden; TTS, total tumor size.

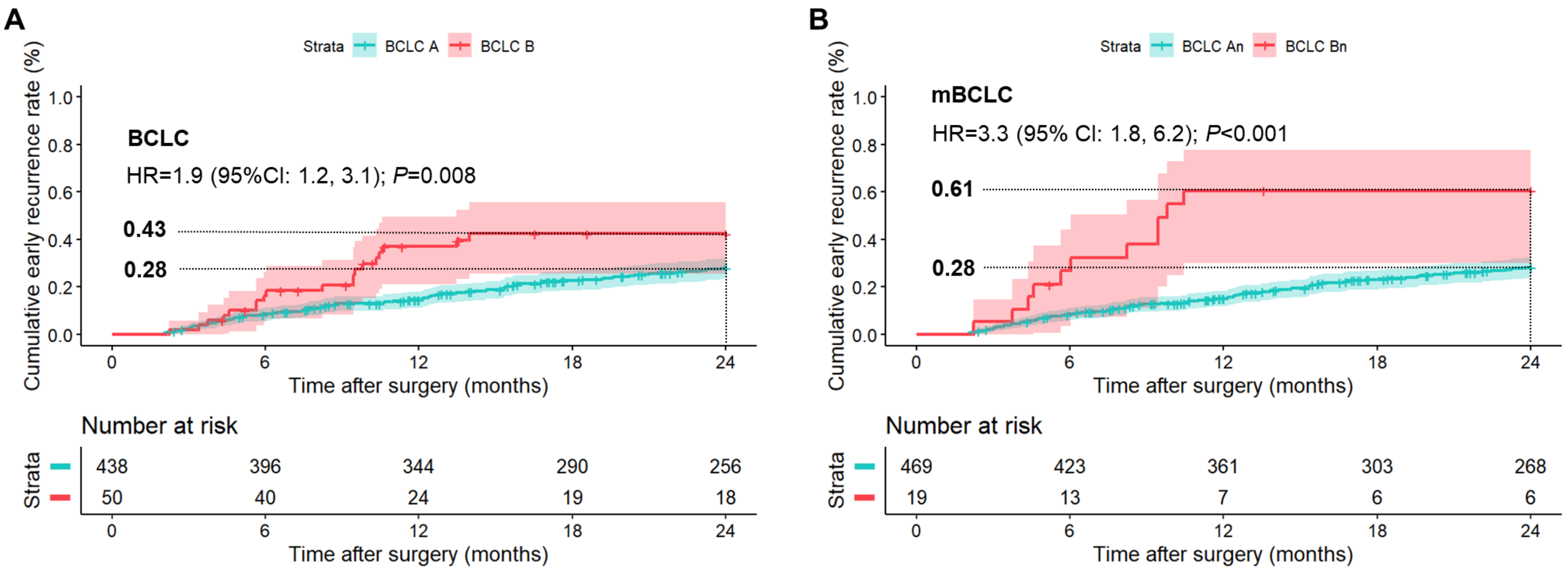

Figure S4 Among patients without adjuvant therapies after surgery, graphs show cumulative rates of early recurrence according to the (**A**) original and (**B**) modified BCLC algorithms. The modified BCLC algorithm provided a greater separation of the cumulative incidence curves compared with the original system. BCLC, Barcelona Clinic Liver Cancer; CI, confidence interval; HR, hazard ratio; mBCLC, modified Barcelona Clinic Liver Cancer.
